# The joint association of insulin resistance index and neutrophil percentage to albumin ratio with acute kidney injury after orthopedic surgery: a retrospective cohort study

**DOI:** 10.3389/fnut.2026.1866149

**Published:** 2026-08-13

**Authors:** Fu Zhang, Xiaomin Hou, Chengwei Tan, Binyan Yin, Lin Bing, Yuqi Zhong, Kexuan Liu, Xinxin Liao, Huamin Liu

**Affiliations:** 1 Department of Anesthesiology, Nanfang Hospital, Southern Medical University, Guangzhou, Guangdong, China; 2 Guangdong Provincial Key Laboratory of Precision Anesthesia and Perioperative Organ Protection, Guangzhou, Guangdong, China; 3 Department of Anesthesiology, Guiyang Second People's Hospital, Guiyang, Guizhou, China; 4 Department of Anesthesiology, Weifang People's Hospital, Weifang, Shandong, China

**Keywords:** acute kidney injury, neutrophil-percentage-to-albumin ratio, orthopedic surgery, risk factor, triglyceride glucose-body mass index

## Abstract

**Background:**

Postoperative acute kidney injury (AKI) is a serious complication following orthopedic surgery. Both insulin resistance (IR) and inflammation have been implicated as risk factors for AKI in surgical populations. This study aimed to evaluate the joint association of IR and inflammatory markers for postoperative AKI after orthopedic surgery.

**Methods:**

This retrospective cohort study enrolled 4,665 adult patients undergoing orthopedic surgery at Nanfang Hospital, Southern Medical University, between January 2018 and October 2023. The primary outcome was AKI; secondary outcomes included intensive care unit (ICU) admission and the length of hospital stay after surgery. IR was evaluated using triglyceride-glucose index (TyG) and TyG-body mass index (TyG-BMI), while inflammatory status was assessed by neutrophil-percentage-to-albumin ratio (NPAR). Multivariable logistic regression models and restricted cubic splines were used to estimate AKI risk. Discriminatory ability was assessed through receiver operating characteristic (ROC) curves with area under curves (AUC). Sensitivity analyses were performed by sequentially excluding predefined high-risk subgroups (e.g., spinal/deformity correction, emergency surgery, diabetes, hyperlipemia, acute trauma/infection) and through a best-case scenario analysis assuming missing creatinine values as non-events.

**Results:**

Among 4,665 patients, 232 (4.97%) developed AKI. In fully adjusted models, both IR indicators and NPAR were linearly associated with an increased risk of AKI. The combination of elevated IR indicators and NPAR demonstrated a significantly stronger association with AKI compared to low levels [odds ratio (OR) and 95% confidence interval (CI): 2.819 (1.749–4.544) for TyG and NPAR; 3.324 (2.022–5.462) for TyG-BMI and NPAR]. The TyG-BMI and NPAR combination yielded superior discrimination ability (AUC: 0.683; 95% CI: 0.648–0.718) compared to either biomarker alone (TyG-BMI AUC: 0.559; NPAR AUC: 0.655). Sensitivity analyses confirmed the robustness of these findings. Additionally, elevated TyG and NPAR were associated with delayed discharge, while elevated TyG-BMI was not. Among the three markers, only NPAR was significantly associated with increased odds of postoperative ICU admission.

**Conclusions:**

Preoperative IR indicators and NPAR are independently associated with AKI after orthopedic surgery. The combination of TyG-BMI and NPAR shows improved discrimination for postoperative AKI. Further prospective multi-center studies are warranted to validate these findings.

## Introduction

Postoperative acute kidney injury (AKI) is a prevalent and serious complication, associated with prolonged hospital stays, increased healthcare costs, progression of chronic kidney disease, and elevated mortality (1, 2). Although postoperative AKI has been extensively investigated in cardiac and major non-cardiac surgeries, its incidence following orthopedic procedures remains inadequately controlled, and the predictive factors in this population warrant further elucidation (3, 4). The pathogenesis of AKI is multifactorial, involving metabolic disturbances, inflammatory stress, hemodynamic instability, and nutritional risk (5, 6).

A key pathophysiological contributor is insulin resistance (IR), which promotes renal injury through mechanisms including oxidative stress, endothelial dysfunction, and chronic low-grade inflammation (7). The triglyceride-glucose index (TyG) and its combined body mass index (BMI) derivative (TyG-BMI) are well-established surrogate markers of IR, and have shown a robust predictive value for cardiovascular-kidney-metabolic disorders (8, 9). Elevated TyG and TyG-BMI have been proven to be associated with an increased risk of AKI in critically ill patients with sepsis (10). However, their association with postoperative AKI, particularly in orthopedic populations, has not yet been established.

Our previous study identified systemic inflammation as a significant contributor to the development of AKI (11). The neutrophil-percentage-to-albumin ratio (NPAR) is a novel and integrative biomarker that reflects both nutritional status and neutrophilic activation, a key component of innate immunity (12). Elevated NPAR has been correlated with adverse outcomes in various immune-mediated inflammatory conditions, including osteoporosis, periodontitis, and breast cancer (13–15). NPAR has also been reported to possess strong discriminatory ability in predicting AKI among patients with liver cirrhosis and ascites (16). In addition, the combination of IR and inflammatory markers has demonstrated valuable joint associations with adverse cardiovascular events, significantly enhancing predictive capacity (17, 18). Nevertheless, whether these indicators improve discrimination ability for AKI following orthopedic surgery remains unexplored.

Given the interconnected nature of metabolic and inflammatory pathways in the pathogenesis of AKI, we hypothesize that the combination of preoperative IR indicators and NPAR may improve discriminative ability for AKI in patients undergoing orthopedic surgery. Accordingly, this retrospective cohort study aimed to evaluate the independent and joint association of IR indicators and NPAR for the occurrence of AKI after orthopedic surgery.

## Methods

### Study design and population

This single-center retrospective cohort study was conducted at Nanfang Hospital, Southern Medical University, Guangdong, China. The study protocol was approved by the Institutional Ethics Committee of Nanfang Hospital, Southern Medical University (Approval number: NFEC-2023-573). A waiver of informed consent was granted owing to the retrospective nature of the study. The study cohort was identified using Perioperative Data Warehouse from Department of Anesthesiology.

**Figure 1:**
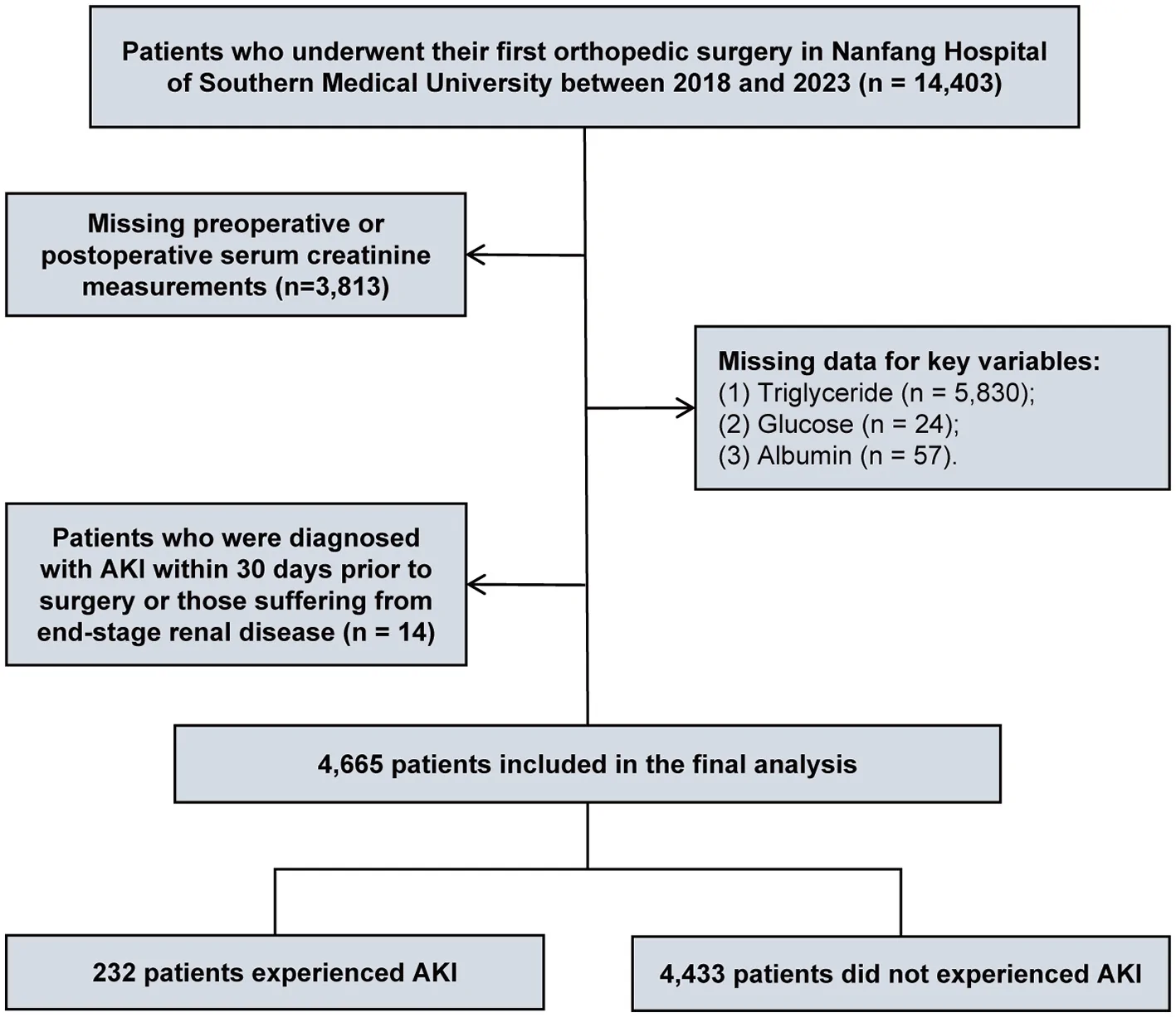
Flowchart of patients' selection.

We screened 14,403 adults who underwent orthopedic surgery between January 2018 and October 2023. For patients with multiple surgical procedures, only the first surgery data was included in the analysis. Exclusion criteria were as follows: (1) Missing preoperative or postoperative (within 7 days) serum creatinine measurements (*n* = 3,813); (2) Missing data for calculating TyG-BMI (*n* = 5,830 for triglycerides, *n* = 24 for glucose) or NPAR (*n* = 57 for albumin); (3) History of AKI within 30 days prior to surgery or end-stage renal disease (defined as kidney transplantation, renal dialysis, or an estimated glomerular filtration rate < 15 mL/min/1.73 m^2^; *n* = 14). The final cohort comprised 4,665 patients (Figure 1).

### Independent variables

Blood glucose, triglycerides, neutrophil percentage, albumin, weight and height were used to calculate independent variables. They were extracted from preoperative laboratory test results closest to surgery date and basic admission information in electronic medical records. Preoperative laboratory test used fasting blood samples, but not necessarily from the same sampling. The formulas were as follows:

- TyG index = Ln [fasting blood glucose (mg/dL) × fasting triglycerides (mg/dL) /2];
- BMI = Weight (kg)/height (m)^2^;
- TyG-BMI index = TyG index × BMI;
- NPAR = neutrophil percentage (%) × 100/albumin (g/dL).

BMI was categorized into underweight (< 18.5 kg/m^2^), normal weight (18.5–23.9 kg/m^2^), overweight (24.0–27.9 kg/m^2^), and obesity (≥28 kg/m^2^) according to the Chinese adult weight classification standard (19).

### Outcomes

The primary outcome was postoperative AKI, defined according to the Kidney Disease: Improving Global Outcomes (KDIGO) serum creatinine criteria: an increase in serum creatinine by ≥0.3 mg/dL within 48 h or a relative increase to ≥1.5 times baseline within 7 days after surgery. The most recent serum creatinine measurement taken within 30 days before surgery was defined as the baseline value. The highest postoperative creatinine value within 7 days after surgery was used to determine AKI. The specific number and proportion of patients who underwent daily serum creatinine measurement after surgery are detailed in Supplementary Table S2. Urine output criteria were not applied owing to the residual effect of intraoperative diuretics in surgical patients.

Secondary outcomes included intensive care unit (ICU) admission after surgery and the length of postoperative hospital stay. ICU admission was defined as unplanned admission to the ICU due to postoperative deterioration (e.g., respiratory failure, hemodynamic instability). Planned admissions for major procedures were not counted as events. The length of postoperative hospital stay was calculated as the time interval between the end of surgery and hospital discharge.

### Covariates

Collected covariates included patient baseline characteristics (age, gender, BMI, smoking and drinking), preoperative comorbidities (diabetes mellitus, hypertension, hyperlipemia, anemia, and heart failure), relevant chronic medications [diuretics, angiotensin-converting enzyme inhibitor/angiotensin II receptor blockers (ACEI/ARB), statins], preoperative laboratory test results [white blood cell (WBC) count, neutrophil percentage, platelet count based on complete blood count, serum creatinine, uric acid (UA), albumin, triglyceride, glucose, high-density lipoprotein cholesterol (HDL-C), sodium, potassium, calcium, estimated glomerular filtration rate (eGFR)], and surgery information [American Society of Anesthesiologists (ASA) physical status, anesthesia method (whether to combine other non-general anesthesia), emergency operation, surgery type, and duration of surgery]. For preoperative laboratory test results, we extracted the most recent measurements taken within 30 days before the patient's surgery date. The preoperative eGFR was calculated using the Chronic Kidney Disease Epidemiology Collaboration equation based on the baseline serum creatinine concentration (20). Baseline renal function was categorized into normal renal function (≥60 mL/min/1.73 m^2^) and impaired renal function (< 60 mL/min/1.73 m^2^) according to the preoperative eGFR.

### Statistical analyses

Continuous variables were presented as mean ± standard deviation (SD) or median (interquartile range, IQR), and compared between groups using Student's *t*-test or Mann-Whitney *U*-test according to the normality. Categorical variables were expressed as frequencies (percentages), and compared using Pearson's Chi-square test.

Logistic regression models were used to assess the risk of AKI, with results reported as odds ratios (OR) and corresponding 95% confidence intervals (CI). TyG, TyG-BMI and NPAR values were categorized into quartiles, with the lowest quartile (Q1) serving as the reference group. Trend tests were performed across quartiles. Standardized transformation of TyG, TyG-BMI and NPAR were conducted to show the effect value for per SD. Three models were constructed: Model 1 was unadjusted; Model 2 adjusted for age, gender, smoking, and drinking. Model 3 further adjusted for diabetes mellitus, hypertension, hyperlipemia, anemia, diuretics, HDL-C, UA, sodium, potassium, calcium, eGFR, emergency, surgery type, anesthesia method, duration of surgery based on Model 2.

Restricted cubic spline (RCS) curves were used to explore potential nonlinear associations of TyG, TyG-BMI, and NPAR with AKI. When linear relationships were identified, the receiver operating characteristic (ROC) curves were generated to evaluate discriminatory ability, and used to determine the optimal cutoffs for TyG, TyG-BMI, and NPAR to define high and low groups for the purpose of additive interaction analysis. The derived cutoffs were not used for risk classification or as a substitute for continuous analyses. TyG and NPAR, as well as TyG-BMI and NPAR, were combined through cross-grouping using a 2 × 2 matrix based on low and high levels. The OR and 95% CI for the group with both high levels were calculated to assess the joint effect of IR indicators and NPAR on AKI occurrence. The improvement in discrimination efficacy was judged through the combined area under ROC (AUC) of IR indicators and NPAR. Comparisons between AUCs were performed using DeLong's test. Additive interaction analyses were conducted to evaluate synergistic effects of TyG or TyG-BMI in combination with NPAR on AKI risk. Additive interaction was quantified using the relative excess risk due to interaction (RERI), attributable proportion (AP), and synergy index (SI), with 95% confidence intervals derived via the delta method according to Hosmer and Lemeshow.

The odds of ICU admission after surgery were examined using logistic regression models. Accelerated failure time models were used to assess the association with the length of postoperative hospital stay (given its right-skewed distribution), reporting time ratios (TR) and 95% CI. Sensitivity analyses were performed by excluding specific patient subgroups, including those undergoing spinal, osteotomy or deformity correction surgery; those undergoing emergency surgery; those with preoperative diabetes mellitus, hypertension, or hyperlipemia; and those with acute trauma or infection (defined as emergency surgery or neutrophil percentage >75%). To further address concerns regarding ascertainment bias, an additional best-case scenario sensitivity analysis was conducted, in which all patients with missing perioperative creatinine measurements (patients with complete exposure indices in the exclusion population) were assumed not to have developed acute kidney injury (excluding those with pre-existing severe renal disease). Missing data (< 10%) were imputed via the random forest algorithm (the missRanger R package). In all analyses, a two-sided *P* < 0.05 indicated statistical significance. All statistical analyses were performed using R software (version 4.4.0; R Foundation for Statistical Computing, Vienna, Austria).

## Results

### Baseline characteristics

A total of 4,665 patients undergoing orthopedic surgery were included in this study. Postoperative AKI occurred in 232 patients (4.97%), of which 190 patients (81.89%) were in stage 1, 27 (11.64%) were in stage 1, and 15 (6.47%) were in stage 3. Compared to the non-AKI group, patients who developed AKI were significantly older [67 (IQR 57, 73) vs. 57 (IQR 45, 67) years] and had a higher prevalence of diabetes mellitus (25.43 vs. 11.17%), hypertension (56.03 vs. 26.93%), anemia (46.98 vs. 20.82%). Laboratory profiles indicated more pronounced inflammatory and metabolic dysregulation in the AKI group, evidenced by significantly higher NPAR, neutrophil percentage, triglyceride and glucose levels, alongside lower albumin and HDL (all *P* < 0.05). Furthermore, the AKI group had a higher proportion of ASA classifications III-IV, emergency operation, and lower proportion of general anesthesia (all *P* < 0.01). The incidence of AKI differed significantly among the surgery types (*P* < 0.001), especially in specific surgical categories like spinal surgery, and osteotomy or deformity correction surgery. ICU admission rates and length of postoperative hospital stay were also significantly elevated in the AKI group (both *P* < 0.001). Additionally, patients with AKI demonstrated significantly higher preoperative levels of TyG [8.83 (8.39, 9.35) vs. 8.62 (8.24, 9.05)], TyG-BMI [216.8 (187.2, 250.4) vs. 208.8 (182.3, 235.9)], and NPAR [18.53 (14.93, 22.65) vs. 15.49 (13.27, 18.84)] compared to the non-AKI patients (Table 1).

**Table 1:** Baseline characteristics of patients with and without AKI.

Characteristics	Total (*n* = 4,665)	Non-AKI (*n* = 4,433)	AKI (*n* = 232)	*P*-value
Female, *n* (%)	2,394 (51.32)	2,279 (51.41)	115 (49.57)	0.584
Age, years	58 (45, 68)	57 (45, 67)	67 (57, 73)	< 0.001
BMI, kg/m^2^	24.1 (21.6, 26.6)	24.1 (21.5, 26.6)	24.7 (21.6, 27.4)	0.089
Smoking, *n* (%)	1,119 (23.99)	1,051 (23.71)	68 (29.31)	0.051
Drinking, *n* (%)	1,060 (22.72)	1,006 (22.69)	54 (23.28)	0.836
Diabetes mellitus, *n* (%)	554 (11.88)	495 (11.17)	59 (25.43)	< 0.001
Hypertension, *n* (%)	1,324 (28.38)	1,194 (26.93)	130 (56.03)	< 0.001
Hyperlipemia, *n* (%)	223 (4.78)	205 (4.62)	18 (7.76)	0.029
Anemia, *n* (%)	1,032 (22.12)	923 (20.82)	109 (46.98)	< 0.001
Heart failure, *n* (%)	5 (0.11)	4 (0.09)	1 (0.43)	0.214
Diuretics, *n* (%)	82 (1.76)	72 (1.62)	10 (4.31)	0.005
ACEI/ARB, *n* (%)	474 (10.16)	441 (9.95)	33 (14.22)	0.036
Statins, *n* (%)	186 (3.99)	171 (3.86)	15 (6.47)	0.048
WBC, × 10^9^	7.18 (5.89, 9.09)	7.16 (5.87, 9.02)	7.74 (6.16, 10.86)	< 0.001
Neutrophil percentage, %	60.9 (53.4, 71.1)	60.7 (53.3, 70.8)	67.0 (56.9, 77.3)	< 0.001
Platelet, × 10^9^	242 (202, 294)	242 (202, 293)	256 (196, 317)	0.201
Creatinine, μmol/L	67 (56, 81)	67 (56, 80)	82 (62, 160)	< 0.001
UA, μmol/L	342 (276, 415)	341 (275, 413)	367 (297, 472)	< 0.001
Albumin, g/dL	3.87 (3.61, 4.11)	3.88 (3.63, 4.12)	3.62 (3.22, 3.89)	< 0.001
Triglyceride, mg/dL	114 (82, 162)	113 (81, 161)	124 (89, 173)	0.043
Glucose, mg/dL	92.5 (83.5, 109.3)	92.2 (83.5, 108.4)	101.8 (85.5, 147.5)	< 0.001
HDL-C, mg/dL	106 (52, 142)	108 (53, 143)	79 (46, 122)	< 0.001
Sodium, mmol/L	141 (139, 142)	141 (139, 142)	141 (139, 142)	0.185
Potassium, mmol/L	3.96 (3.76, 4.21)	3.96 (3.75, 4.20)	4.16 (3.85, 4.64)	< 0.001
Calcium, mmol/L	2.27 (2.19, 2.34)	2.27 (2.19, 2.34)	2.24 (2.17, 2.34)	0.172
eGFR, *n* (%)				< 0.001
≥60 mL/min/1.73 m^2^	3,596 (77.09)	3,498 (78.91)	98 (42.24)	
< 60 mL/min/1.73 m^2^	1,069 (22.91)	935 (21.09)	134 (57.76)	
ASA, *n* (%)				< 0.001
1	658 (14.11)	651 (14.69)	7 (3.02)	
2	3,194 (68.47)	3,068 (69.21)	126 (54.31)	
3	769 (16.48)	681 (15.36)	88 (37.93)	
4	44 (0.94)	33 (0.74)	11 (4.74)	
Anesthesia method, *n* (%)				< 0.001
General anesthesia	2,828 (60.62)	2,728 (61.54)	100 (43.10)	
Combine other anesthesia method	1,837 (39.38)	1,705 (38.46)	132 (56.90)	
Emergency, *n* (%)	139 (2.98)	125 (2.82)	14 (6.03)	0.005
Surgery type, *n* (%)				< 0.001
Arthroplasty	1,433 (30.72)	1,346 (30.36)	87 (37.50)	
Open reduction and internal fixation	842 (18.05)	813 (18.34)	29 (12.50)	
Spinal surgery	989 (21.20)	975 (21.99)	14 (6.03)	
Osteotomy or deformity correction	166 (3.56)	146 (3.29)	20 (8.62)	
Bone lesion or soft tissue resection surgery	409 (8.77)	378 (8.53)	31 (13.36)	
Others	826 (17.71)	775 (17.48)	51 (21.98)	
Duration of surgery, *n* (%)				0.483
< 60 min	331 (7.10)	313 (7.06)	18 (7.76)	
≥60 and < 150 min	2,634 (56.46)	2,496 (56.30)	138 (59.48)	
≥150 min	1,700 (36.44)	1,624 (36.63)	76 (32.76)	
Length of postoperative hospital stay, days	10 (8, 14)	10 (8, 14)	12 (9, 19)	< 0.001
ICU admission, *n* (%)	221 (4.74)	199 (4.49)	22 (9.48)	< 0.001
Stage of AKI, *n* (%)				—
Stage 1	190 (4.07)	—	190 (81.89)	
Stage 2	27 (0.58)	—	27 (11.64)	
Stage 3	15 (0.32)	—	15 (6.47)	
TyG	8.63 (8.25, 9.06)	8.62 (8.24, 9.05)	8.83 (8.39, 9.35)	< 0.001
TyG-BMI	209.2 (182.6, 236.6)	208.8 (182.3, 235.9)	216.8 (187.2, 250.4)	0.003
NPAR	15.58 (13.34, 19.09)	15.49 (13.27, 18.84)	18.53 (14.93, 22.65)	< 0.001

ACEI/ARB, angiotensin converting enzyme inhibitor/angiotensin II receptor blockers; AKI, acute kidney injury; ASA, American Society of Anesthesiologists; BMI, body mass index; eGFR, estimated glomerular filtration rate; HDL-C, high-density lipoprotein cholesterol; ICU, intensive care unit; NPAR, neutrophil percentage to albumin ratio; TyG, triglyceride-glucose; TyG-BMI, triglyceride glucose-body mass index; UA, uric acid; WBC, white blood cell.

However, compared with excluded patients, the analytic cohort were more likely to have high-risk properties, such as older age, greater proportion of preoperative comorbidities (diabetes mellitus, hypertension, and anemia), higher inflammation status (Supplementary Table S1).

### Independent associations of TyG, TyG-BMI, and NPAR with AKI

**Figure 2:**
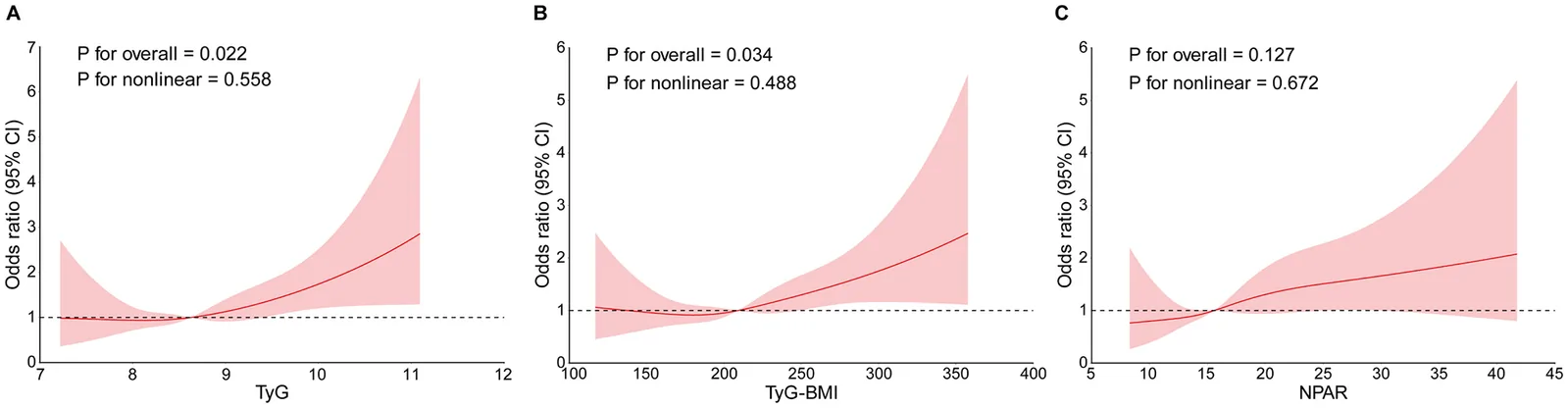
RCS curve between TyG, TyG-BMI, and NPAR and the risk of AKI. **(A)** TyG and AKI. **(B)** TyG-BMI and AKI. **(C)** NPAR and AKI. AKI, acute kidney injury; NPAR, neutrophil percentage to albumin ratio; RCS, restricted cubic spline; TyG, triglyceride-glucose; TyG-BMI, triglyceride glucose-body mass index. All analyses were adjusted for confounding factors including age, gender, smoking, drinking, diabetes mellitus, hypertension, hyperlipemia, anemia, diuretics, HDL-C, UA, sodium, potassium, calcium, eGFR, emergency, surgery type, anesthesia method, duration of surgery.

In the unadjusted model (Model 1), higher quartiles of TyG, TyG-BMI, and NPAR were significantly associated with increased incidence and risk of AKI, showing significant trends (all *P* for trend < 0.01). The increased risk persisted after full adjustment for demographic, clinical, and surgical factors (Model 3). Per SD increase, the adjusted ORs were 1.249 (95% CI, 1.088–1.434) for TyG, 1.223 (95% CI, 1.067–1.403) for TyG-BMI, and 1.210 (95% CI, 1.044–1.404) for NPAR. The highest quartile (Q4) of TyG (adjusted OR, 1.568, 95% CI, 1.026–2.395), and TyG-BMI (adjusted OR, 1.656, 95% CI, 1.108–2.474) contributed to a significantly elevated AKI risk compared to those in the lowest quartile (Q1; Table 2). Restricted cubic spline analysis confirmed linear dose-response relationships between these indices and AKI risk (all *P* for nonlinearity >0.05; Figure 2).

**Table 2:** Logistic regression analyses for the association of TyG, TyG-BMI, and NPAR with AKI.

Variables	AKI (%)	Model 1	*P*-value	Model 2	*P-*value	Model 3	*P*-value
		OR (95% CI)		OR (95% CI)		OR (95% CI)	
TyG							
Q1	40 (3.43)^a^	Reference		Reference		Reference	
Q2	46 (3.95)^a, b^	1.156 (0.751, 1.780)	0.510	1.058 (0.683, 1.637)	0.802	1.027 (0.654, 1.612)	0.909
Q3	64 (5.49)^b, c^	1.635 (1.092, 2.448)	0.017	1.422 (0.945, 2.141)	0.091	1.389 (0.906, 2.128)	0.132
Q4	82 (7.03)^c^	2.127 (1.445, 3.133)	< 0.001	1.928 (1.302, 2.856)	0.001	1.568 (1.026, 2.395)	0.038
*P* for trend		1.305 (1.156, 1.473)	< 0.001	1.269 (1.036, 1.058)	< 0.001	1.183 (1.035, 1.352)	0.013
Per SD		1.384 (1.226, 1.562)	< 0.001	1.341 (1.183, 1.519)	< 0.001	1.249 (1.088, 1.434)	0.002
TyG-BMI							
Q1	50 (4.29)^a^	Reference		Reference		Reference	
Q2	51 (4.37)^a^	1.021 (0.685, 1.521)	0.919	0.974 (0.650, 1.460)	0.898	1.023 (0.669, 1.565)	0.916
Q3	50 (4.29)^a^	1.000 (0.670, 1.493)	1.000	0.928 (0.618, 1.393)	0.719	0.998 (0.649, 1.534)	0.991
Q4	81 (6.94)^b^	1.665 (1.159, 2.391)	0.006	1.693 (1.168, 2.455)	0.005	1.656 (1.108, 2.474)	0.014
*P* for trend		1.183 (1.050, 1.333)	0.004	1.190 (1.052, 1.347)	< 0.001	1.180 (1.034, 1.345)	0.014
Per SD		1.235 (1.089, 1.401)	0.001	1.254 (1.100, 1.430)	< 0.001	1.223 (1.067, 1.403)	0.004
NPAR							
Q1	30 (2.57)^a^	Reference		Reference		Reference	
Q2	39 (3.34)^a, b^	1.310 (0.808, 2.124)	0.273	1.171 (0.721, 1.902)	0.524	1.075 (0.656, 1.764)	0.773
Q3	56 (4.80)^b^	1.910 (1.217, 2.999)	0.005	1.545 (0.978, 2.441)	0.062	1.297 (0.808, 2.082)	0.281
Q4	107 (9.17)^c^	3.822 (2.528, 5.780)	< 0.001	2.522 (1.637, 3.885)	< 0.001	1.641 (0.998, 2.699)	0.051
*P* for trend		1.610 (1.416, 1.831)	< 0.001	1.393 (1.217, 1.594)	< 0.001	1.189 (1.014, 1.394)	0.033
Per SD		1.579 (1.428, 1.746)	< 0.001	1.421 (1.272, 1.588)	< 0.001	1.210 (1.044, 1.404)	0.012

AKI, acute kidney injury; CI, confidence interval; NPAR, neutrophil percentage to albumin ratio; OR, odds ratio; SD, standard deviation; TyG, triglyceride-glucose; TyG-BMI, triglyceride glucose-body mass index.

^a, b, c^The same superscript letters indicate the AKI incidence was no significant difference among the quartiles.

Model 1: unadjusted.

Model 2: adjusted for age, gender, smoking, drinking.

Model 3: further adjusted for diabetes mellitus, hypertension, hyperlipemia, anemia, diuretics, HDL-C, UA, sodium, potassium, calcium, eGFR, emergency, surgery type, anesthesia method, duration of surgery based on Model 2.

### Discriminatory ability of TyG, TyG-BMI and NPAR for AKI

**Figure 3:**
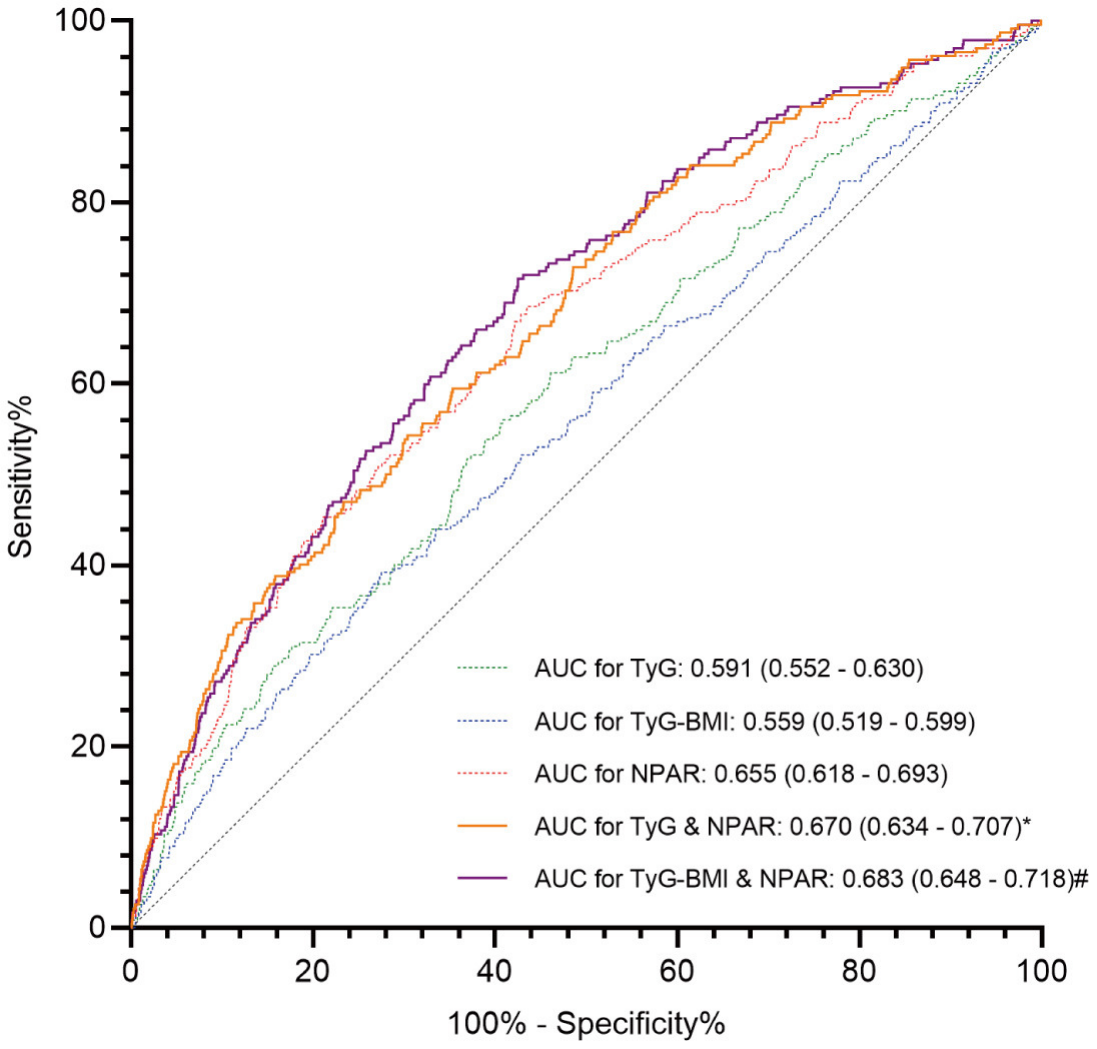
ROC curves between TyG, TyG-BMI, NPAR, TyG & NPAR, TyG_BMI & NPAR, and AKI. *The AUC was significantly higher than that of TyG alone. ^#^The AUC was significantly higher than that of TyG-BMI and NPAR alone. AKI, acute kidney injury; AUC, area under the curve; NPAR, neutrophil percentage to albumin ratio; ROC, receiver operating characteristic; TyG, triglyceride-glucose; TyG-BMI, triglyceride glucose-body mass index.

ROC curve analysis assessed the discriminative ability of these indices. NPAR alone showed the highest AUC among the single indicators (AUC, 0.655, 95% CI, 0.618–0.693). The combined indices demonstrated better discrimination ability: TyG-BMI combined with NPAR achieved the highest AUC of 0.683 (95% CI, 0.648–0.718), which was significantly higher than that of TyG-BMI or NPAR alone. Similarly, the combination of TyG and NPAR had an AUC of 0.670 (95% CI, 0.634–0.707), which was superior to TyG alone (Figure 3 and Supplementary Table S3).

### Joint associations of insulin resistance indicators and NPAR on AKI risk

When TyG and NPAR were combined into a 2 × 2 matrix based on cutoff values in ROC curves, patients with high levels of both markers had the highest risk of AKI (adjusted OR, 2.819, 95% CI, 1.749–4.544). Similarly, the combination of high TyG-BMI and high NPAR was associated with a substantially elevated risk (adjusted OR, 3.324, 95% CI, 2.022–5.462; Table 3). Additive interaction between TyG and NPAR on AKI risk was observed in unadjusted and partially adjusted models (Model 1 and 2), but the additive effect was attenuated to non-significance (RERI: 0.653, 95% CI: −0.381 to 1.624) after full adjustment (Model 3), with no evidence of interaction between TyG-BMI and NPAR across all models (Supplementary Tables S4, S5).

**Table 3:** Logistic regression analyses for the association of TyG combined with NPAR, TyG-BMI combined with NPAR, with AKI.

Variables	Model 1	*P*-value	Model 2	*P*-value	Model 3	*P*-value
	OR (95% CI)		OR (95% CI)		OR (95% CI)	
TyG combined with NPAR						
TyG low level and NPAR low level	Reference		Reference		Reference	
TyG high level and NPAR low level	1.850 (1.154, 2.965)	0.010	1.626 (1.014, 2.607)	0.044	1.507 (0.926, 2.451)	0.098
TyG low level and NPAR high level	2.812 (1.830, 4.321)	< 0.001	2.002 (1.303, 3.075)	0.002	1.658 (1.034, 2.658)	0.036
TyG high level and NPAR high level	5.447 (3.577, 8.293)	< 0.001	3.877 (2.546, 5.902)	< 0.001	2.819 (1.749, 4.544)	< 0.001
TyG-BMI combined with NPAR						
TyG-BMI low level and NPAR low level	Reference		Reference		Reference	
TyG-BMI high level and NPAR low level	2.748 (1.721, 4.288)	< 0.001	2.547 (1.595, 4.067)	< 0.001	2.159 (1.326, 3.516)	0.002
TyG-BMI low level and NPAR high level	3.865 (2.587, 5.776)	< 0.001	2.784 (1.863, 4.161)	< 0.001	2.075 (1.323, 3.252)	0.002
TyG-BMI high level and NPAR high level	6.278 (3.981, 9.899)	< 0.001	4.855 (3.079, 7.656)	< 0.001	3.324 (2.022, 5.462)	< 0.001

AKI, acute kidney injury; CI, confidence interval; NPAR, neutrophil percentage to albumin ratio; OR, odds ratio; TyG, triglyceride-glucose; TyG-BMI, triglyceride glucose-body mass index. Patients were classified into two categories (low level or high level) based on cutoff value of variables.

Model 1: unadjusted.

Model 2: adjusted for age, gender, smoking, drinking.

Model 3: further adjusted for diabetes mellitus, hypertension, hyperlipemia, anemia, diuretics, HDL-C, UA, sodium, potassium, calcium, eGFR, emergency, surgery type, general anesthesia, duration of surgery based on Model 2.

### Sensitivity analyses for primary outcome

The robustness of the primary findings was confirmed through excluding specific patient subgroups. The significant associations of TyG, TyG-BMI, NPAR, and their combinations with AKI risk persisted, with largely consistent effect sizes, after sequentially excluding patients who underwent specific surgery types including spinal and osteotomy/deformity correction (Supplementary Tables S6, S7), those with pre-existing diabetes mellitus (Supplementary Tables S8, S9), hyperlipidemia (Supplementary Tables S10, S11), or emergency surgery (Supplementary Tables S12, S13), and those with emergency surgery or neutrophil percentage >75% (Supplementary Tables S14, S15). Given the higher-risk profile as shown in Supplementary Table S1, which raised concerns about potential ascertainment bias, we additionally performed a best-case scenario sensitivity analysis including patients with complete exposure indices in the exclusion population. The results of this analysis were consistent with those of our primary analysis, supporting the robustness of our findings (Supplementary Tables S16, S17). These findings suggest that our main results are unlikely to be substantially affected by ascertainment bias. The linear dose-response relationships of TyG, TyG-BMI, and NPAR with AKI risk persisted in these sensitivity analysis (all *P* for nonlinearity >0.05; Supplementary Figures S1–S6). ROC analyses in these sensitivity analyses consistently showed that the combined biomarkers maintained better discrimination ability than the individual components (Supplementary Tables S18–S23, Supplementary Figures S7–S12).

### Secondary outcomes and exploratory analyses

In secondary analyses, higher levels of TyG and NPAR were significantly associated with an increased length of postoperative hospital stay [adjusted TR and 95% CI: 1.038 (1.020, 1.056) for TyG per SD; 1.196 (1.172, 1.221) for NPAR per SD]. However, TyG-BMI was negatively associated with the length of postoperative hospital stay [adjusted TR and 95% CI: 0.986 (0.971, 1.003)]. We explored whether the introduction of BMI in the TyG-BMI formula accounted for this inverse association. As expected, overweight was associated with a shorter length of postoperative hospital stay [adjusted TR and 95% CI: 0.951 (0.917, 0.986)]. In contrast, although underweight patients showed a prolonged length of stay, the association did not reach statistical significance [adjusted TR and 95% CI: 1.032 (0.965, 1.104)]. The combination of high TyG and NPAR was associated with a longer postoperative hospital stay [adjusted TR and 95% CI: 1.323 (1.256, 1.394)], and the combined group of high TyG-BMI and NPAR also had this association [adjusted TR and 95% CI: 1.189 (1.121, 1.261); Supplementary Table S24].

Additionally, NPAR was independently associated with a higher risk of ICU admission (Q4 vs. Q1: OR, 5.209, 95% CI, 3.141–8.638), whereas TyG and TyG-BMI showed no significant association with this outcome (Supplementary Table S25). Nevertheless, the combination of high TyG and NPAR, as well as high TyG-BMI and NPAR, was associated with the highest odds of ICU admission [adjusted OR and 95% CI, 3.493 (2.234, 5.460) and 3.432 (2.155, 5.465); Supplementary Table S26]. Conversely, obese patients had a higher odds of AKI [adjusted OR and 95% CI: 1.763 (1.167–2.582)] relative to normal weight patients (Supplementary Table S27).

## Discussion

In this retrospective study, we found that higher preoperative TyG, TyG-BMI, and NPAR were independently associated with an increased risk of AKI. The combination of higher TyG or TyG-BMI with higher NPAR demonstrated a stronger association with AKI compared to either marker alone. Notably, the combination of TyG-BMI and NPAR exhibited superior discrimination ability for AKI occurrence. Additionally, higher TyG and NPAR were associated with prolonged postoperative hospital stay. Only NPAR was significantly associated with increased odds of ICU admission following surgery.

Postoperative AKI following orthopedic procedures is a well-recognized complication with a multifactorial etiology, encompassing metabolic stress, systemic inflammation, hemodynamic perturbations, and exposure to nephrotoxic agents (21, 22). Specific risk factors include advanced age, pre-existing renal impairment, diabetes mellitus, hypertension, intraoperative blood loss, hypotension, and the use of nephrotoxic drugs such as non-steroidal anti-inflammatory drugs (NSAIDs) or contrast agents (23, 24). The reported incidence of AKI after orthopedic surgery ranges from 6.7 to 10.8% (25). In particular, large-scale joint replacements and spinal surgeries are associated with substantial blood loss, prolonged operative time, and the potential release of inflammatory mediators from bone cement, all of which may contribute to an elevated risk of AKI (26, 27). Despite this, identifying patients at heightened preoperative risk remains a clinical challenge.

TyG-BMI is a composite indicator that integrates IR (via the TyG index) and adiposity (via BMI), offering a robust surrogate of metabolic dysregulation (28). While prior research has largely focused on its association with long-term cardiovascular-kidney-metabolic outcomes, including chronic kidney disease (CKD), cardiovascular disease, and stroke, our study extends its relevance to acute postoperative renal injury (8, 29). A longitudinal cohort study has shown that cumulative average TyG-BMI was independently associated with the risk of CKD (30). Elevated TyG-BMI reflects aggravated IR, which predisposes individuals to hyperglycemia and hyperlipemia (31). Hyperglycemia promotes renal tubular injury through formation of advanced glycation end products and increased oxidative stress, while elevated free fatty acids exert direct lipotoxic effects on tubular cells (32, 33). IR further amplifies renal inflammation by upregulating pro-inflammatory cytokines and activating signaling pathways such as NF-κB, thereby promoting fibrosis and functional decline (34, 35). These pathways provide a plausible biological link between preoperative metabolic status and susceptibility to perioperative AKI.

NPAR simultaneously reflects innate immune function and nutrition status (36). Elevated NPAR indicates increased neutrophil activity or decreased albumin, the latter of which is associated with impaired antioxidant capacity, endothelial dysfunction, and heightened systemic inflammatory tone (37, 38). Neutrophil infiltration leads to the release of reactive oxygen species (ROS) and inflammatory mediators (e.g., interleukine-1β, interleukine-6), directly damaging renal tubules and amplifying local inflammation (39). Clinically, NPAR has shown prognostic value in conditions such as sepsis, heart failure, and diabetic kidney disease, supporting its association with inflammation-driven organ injury (40–42). Our findings align with and extend this body of evidence, identifying NPAR as a preoperative indicator of AKI in surgical patients.

Our study revealed improved discrimination ability achieved by combining TyG-BMI and NPAR, which yield a significantly higher AUC than that of TyG-BMI and NPAR alone, likely attributable to their representation of complementary pathological axes. IR and hyperlipemia induce endothelial dysfunction and oxidative stress, thereby priming the kidney for inflammatory injury (43). Systemic inflammation (captured by NPAR) can, in turn, exacerbate metabolic dysregulation, creating a vicious cycle that heightens renal susceptibility during the perioperative period (44). This bidirectional crosstalk between metabolic and inflammatory pathways provides a compelling rationale for their combined application in risk discrimination. Our results are consistent with emerging evidence indicating that multimodal biomarkers improve discrimination ability for AKI compared to single-parameter models (45). Nevertheless, the combination of TyG and NPAR did not yield a significantly higher AUC compared to the use of NPAR alone, underscoring the enhanced clinical utility of TyG-BMI relative to TyG. Given the modest AUC values, the clinical utility of these biomarkers remains limited at present, and their standalone application in decision-making is not warranted. Future prospective studies with larger cohorts are needed to determine whether adding these biomarkers to established clinical risk scores could offer incremental discriminatory value.

Interestingly, both TyG and NPAR were significantly associated with longer postoperative hospital stay, whereas TyG-BMI tended to be negatively associated with postoperative hospital stay, although this association disappeared in the fully adjusted model. We explored whether the introduction of BMI into the TyG-BMI formula accounted for this divergence. As expected, higher BMI as overweight was associated with a shorter postoperative hospital stay. This observation also reflects the so-called “obesity paradox,” suggesting that overweight or obese patients may experience better recovery outcomes in selected surgical populations, a phenomenon documented in other clinical contexts (46). However, this speculative interpretation remains exploratory and cannot be conclusively established from our current data, warranting further validation in prospective studies.

Several limitations of this study should be acknowledged. First, the retrospective design at a single center may introduce potential unmeasured confounding and limit generalizability of our conclusions. Second, AKI diagnosis was based solely on serum creatinine criteria due to the unavailability of urine output data, possibly underestimating the true incidence of AKI. Third, the exclusion of patients lacking postoperative creatinine measurements or the laboratory data required to calculate the exposures may have introduced selection bias. Fourth, the unavailability of other perioperative factors (e.g., intraoperative blood loss, transfusion, hypotension, vasopressor use, fluid balance, and postoperative nephrotoxic medications) precludes adjustment for these variables, leaving residual confounding possible. Finally, external validation across diverse surgical populations and settings is necessary prior to clinical implementation.

## Conclusion

In conclusion, preoperative TyG-BMI and NPAR are independent and combinable risk factors of AKI after orthopedic surgery. The integration of these readily available biomarkers improves discriminatory ability for postoperative AKI, facilitating the early identification of high-risk patients. This approach supports targeted preoperative assessment and underscores the intertwined roles of metabolic and inflammatory pathways in the pathogenesis of postoperative renal injury. Further research should focus on prospective validation of this model and investigation of its utility in guiding preventive strategies.

## Data Availability

The raw data supporting the conclusions of this article will be made available by the authors, without undue reservation.
