# Supplementary material for: The joint association of insulin resistance index and neutrophil percentage to albumin ratio with acute kidney injury after orthopedic surgery: a retrospective cohort study
Source: Front Nutr. 2026 Aug 13;13:1866149. doi: 10.3389/fnut.2026.1866149 (PMC13518200; doi:10.3389/fnut.2026.1866149)
Supplement: Supplementary file 1 [file Table_1.docx]

**The Joint Association of Insulin Resistance Index and Neutrophil Percentage to Albumin Ratio with Acute Kidney Injury After Orthopedic Surgery: A Retrospective Cohort Study**

**Supplementary Table legends**

**Table S1** Baseline characteristics of included patients versus those excluded

**Table S2** The number and percentage of patients with daily creatinine measurements after operation

**Table S3** Predictive ability of TyG, TyG-BMI, NPAR, TyG & NPAR, TyG-BMI & NPAR for AKI

**Table S4** Additive interaction between TyG and NPAR on AKI risk

**Table S5** Additive interaction between TyG-BMI and NPAR on AKI risk

**Table S6** Sensitivity analysis for the association of TyG, TyG-BMI, and NPAR with AKI after excluding spinal, osteotomy or deformity correction surgery

**Table S7** Sensitivity analysis for the association of TyG/TyG-BMI combined with NPAR with AKI after excluding spinal, osteotomy or deformity correction surgery

**Table S8** Sensitivity analysis for the association of TyG, TyG-BMI, and NPAR with AKI after excluding patients with diabetes

**Table S9** Sensitivity analysis for the association of TyG/TyG-BMI combined with NPAR with AKI after excluding patients with diabetes

**Table S10** Sensitivity analysis for the association of TyG, TyG-BMI, and NPAR with AKI after excluding patients with hyperlipemia

**Table S11** Sensitivity analysis for the association of TyG/TyG-BMI combined with NPAR with AKI after excluding patients with hyperlipemia

**Table S12** Sensitivity analysis for the association of TyG, TyG-BMI, and NPAR with AKI after excluding patients with emergency surgery

**Table S13** Sensitivity analysis for the association of TyG/TyG-BMI combined with NPAR with AKI after excluding patients with emergency surgery

**Table S14** Sensitivity analysis for the association of TyG, TyG-BMI, and NPAR with AKI after excluding patients with emergency surgery and neutrophil percentage > 75%

**Table S15** Sensitivity analysis for the association of TyG/TyG-BMI combined with NPAR with AKI after excluding patients with emergency surgery and neutrophil percentage > 75%

**Table S16** Sensitivity analysis for the association of TyG, TyG-BMI, and NPAR with AKI after including patients with complete exposure indices in the exclusion population

**Table S17** Sensitivity analysis for the association of TyG/TyG-BMI combined with NPAR with AKI after including patients with complete exposure indices in the exclusion population

**Table S18** Sensitivity analysis for predictive ability of TyG, TyG-BMI, NPAR, TyG/TyG-BMI combined with NPAR for AKI after excluding spinal, osteotomy or deformity correction surgery

**Table S19** Sensitivity analysis for predictive ability of TyG, TyG-BMI, NPAR, TyG/TyG-BMI combined with NPAR for AKI after excluding patients with diabetes

**Table S20** Sensitivity analysis for predictive ability of TyG, TyG-BMI, NPAR, TyG/TyG-BMI combined with NPAR for AKI after excluding patients with hyperlipemia

**Table S21** Sensitivity analysis for predictive ability of TyG, TyG-BMI, NPAR, TyG/TyG-BMI combined with NPAR for AKI after excluding patients with emergency surgery

**Table S22** Sensitivity analysis for predictive ability of TyG, TyG-BMI, NPAR, TyG/TyG-BMI combined with NPAR for AKI after excluding patients with emergency surgery and neutrophil percentage > 75%

**Table S23** Sensitivity analysis for predictive ability of TyG, TyG-BMI, NPAR, TyG/TyG-BMI combined with NPAR for AKI after including patients with complete exposure indices in the exclusion population

**Table S24** Accelerated failure time model of TyG, TyG-BMI, NPAR, TyG/TyG-BMI combined with NPAR with the length of postoperative hospital stay

**Table S25** Logistic regression analysis for the association of TyG, TyG-BMI, and NPAR with ICU admission

**Table S26** Logistic regression analysis for the association of TyG/TyG-BMI combined with NPAR with ICU admission

**Table S27** Association of BMI with postoperative AKI

**Supplementary Figure legends**

**Figure S1** Sensitivity analysis for RCS curve between TyG, TyG-BMI, and NPAR and the risk of AKI after excluding spinal, osteotomy or deformity correction surgery

**Figure S2** Sensitivity analysis for RCS curve between TyG, TyG-BMI, and NPAR and the risk of AKI after excluding patients with diabetes

**Figure S3** Sensitivity analysis for RCS curve between TyG, TyG-BMI, and NPAR and the risk of AKI after excluding patients with hyperlipemia

**Figure S4** Sensitivity analysis for RCS curve between TyG, TyG-BMI, and NPAR and the risk of AKI after excluding patients with emergency surgery

**Figure S5** Sensitivity analysis for RCS curve between TyG, TyG-BMI, and NPAR and the risk of AKI after excluding patients with emergency surgery and neutrophil percentage > 75%

**Figure S6** Sensitivity analysis for RCS curve between TyG, TyG-BMI, and NPAR and the risk of AKI after including patients with complete exposure indices in the exclusion population

**Figure S7** Sensitivity analysis for ROC curves between TyG, TyG-BMI, NPAR, TyG/TyG-BMI combined with NPAR and AKI after excluding spinal, osteotomy or deformity correction surgery

**Figure S8** Sensitivity analysis for ROC curves between TyG, TyG-BMI, NPAR, TyG/TyG-BMI combined with NPAR and AKI after excluding patients with diabetes

**Figure S9** Sensitivity analysis for ROC curves between TyG, TyG-BMI, NPAR, TyG/TyG-BMI combined with NPAR and AKI after excluding patients with hyperlipemia

**Figure S10** Sensitivity analysis for ROC curves between TyG, TyG-BMI, NPAR, TyG/TyG-BMI combined with NPAR and AKI after excluding patients with emergency surgery

**Figure S11** Sensitivity analysis for ROC curves between TyG, TyG-BMI, NPAR, TyG/TyG-BMI combined with NPAR and AKI after excluding patients with emergency surgery and neutrophil percentage > 75%

**Figure S12** Sensitivity analysis for ROC curves between TyG, TyG-BMI, NPAR, TyG/TyG-BMI combined with NPAR and AKI after including patients with complete exposure indices in the exclusion population

**Table S1** Baseline characteristics of included patients versus those excluded

| **Characteristics** | **Total**  **(n = 14,403)** | **Included**  **(n = 4,665)** | **Excluded**  **(n = 9,738)** | ***P* value** |
| --- | --- | --- | --- | --- |
| Female, n (%) | 6725 (46.69) | 2394 (51.32) | 4331 (44.48) | <0.001 |
| Age, years | 53 (41, 63) | 58 (45, 68) | 51 (40, 60) | <0.001 |
| BMI, kg/m^2^ | 24.0 (21.7, 26.4) | 24.1 (21.6, 26.6) | 23.9 (21.7, 26.2) | 0.098 |
| Smoking, n (%) | 3397 (24.05) | 1119 (23.99) | 2278 (24.09) | 0.895 |
| Drinking, n (%) | 3289 (23.51) | 1060 (22.72) | 2229 (23.91) | 0.120 |
| Diabetes mellitus, n (%) | 1354 (9.40) | 554 (11.88) | 800 (8.22) | <0.001 |
| Hypertension, n (%) | 2916 (20.25) | 1324 (28.38) | 1592 (16.35) | <0.001 |
| Hyperlipemia, n (%) | 757 (5.26) | 223 (4.78) | 534 (5.48) | 0.077 |
| Anemia, n (%) | 1995 (13.85) | 1032 (22.12) | 963 (9.89) | <0.001 |
| Heart failure, n (%) | 9 (0.06) | 5 (0.11) | 4 (0.04) | 0.259 |
| Diuretics, n (%) | 148 (1.03) | 82 (1.76) | 66 (0.68) | <0.001 |
| ACEI/ARB, n (%) | 950 (6.60) | 474 (10.16) | 476 (4.89) | <0.001 |
| Statins, n (%) | 638 (4.43) | 186 (3.99) | 452 (4.64) | 0.074 |
| WBC, ×10^9^ | 6.86 (5.64, 8.47) | 7.18 (5.89, 9.09) | 6.70 (5.56, 8.23) | <0.001 |
| Neutrophil percentage, % | 58.5 (51.8, 66.8) | 60.9 (53.4, 71.1) | 57.6 (51.1, 65.0) | <0.001 |
| Platelet, ×10^9^ | 237 (199, 282) | 242 (202, 294) | 234 (198, 276) | <0.001 |
| Creatinine, µmol/L | 69 (58, 82) | 67 (56, 81) | 69 (58, 82) | <0.001 |
| UA, µmol/L | 343 (279, 414) | 342 (276, 415) | 344 (281, 413) | 0.275 |
| Albumin, g/dL | 3.96 (3.74, 4.21) | 3.87 (3.61, 4.11) | 4.01 (3.79, 4.25) | <0.001 |
| Triglyceride, mg/dl | 113 (82, 163) | 114 (82, 162) | 113 (81, 164) | 0.855 |
| Glucose, mg/dl | 90.2 (82.4, 102.4) | 92.5 (83.5, 109.3) | 89.3 (81.9, 99.7) | <0.001 |
| HDL-C, mg/dl | 53 (42, 113) | 106 (52, 142) | 44 (38, 51) | <0.001 |
| Sodium, mmol/L | 141 (139, 142) | 141 (139, 142) | 141 (139, 142) | 0.181 |
| Potassium, mmol/L | 3.94 (3.74, 4.16) | 3.96 (3.76, 4.21) | 3.93 (3.74, 4.14) | <0.001 |
| Calcium, mmol/L | 2.28 (2.21, 2.35) | 2.27 (2.19, 2.34) | 2.29 (2.22, 2.35) | <0.001 |
| eGFR, n (%) |  |  |  | <0.001 |
| ≥60 mL/min/1.73m^2^ | 11844 (82.48) | 3596 (77.09) | 8248 (85.08) |  |
| <60 mL/min/1.73m^2^ | 2516 (17.52) | 1069 (22.91) | 1447 (14.92) |  |
| ASA, n (%) |  |  |  | <0.001 |
| 1 | 3217 (22.95) | 658 (14.11) | 2559 (27.36) |  |
| 2 | 9529 (67.98) | 3194 (68.47) | 6335 (67.74) |  |
| 3 | 1215 (8.67) | 769 (16.48) | 446 (4.77) |  |
| ~~4~~ | 56 (0.40) | 44 (0.94) | 12 (0.13) |  |
| Anesthesia method, n (%) |  |  |  | <0.001 |
| General anesthesia | 8882 (63.35) | 2828 (60.62) | 6054 (64.71) |  |
| Combine other anesthesia method | 5139 (36.65) | 1837 (39.38) | 3302 (35.29) |  |
| Emergency, n (%) | 990 (6.87) | 139 (2.98) | 851 (8.74) | <0.001 |
| Surgery type, n (%) |  |  |  | <0.001 |
| Arthroplasty | 3460 (24.02) | 1433 (30.72) | 2027 (20.82) |  |
| Open reduction and internal fixation | 1924 (13.36) | 842 (18.05) | 1082 (11.11) |  |
| Spinal surgery | 5792 (40.21) | 989 (21.20) | 4803 (49.32) |  |
| Osteotomy or deformity correction | 310 (2.15) | 166 (3.56) | 144 (1.48) |  |
| Bone lesion or soft tissue resection surgery | 1362 (9.46) | 409 (8.77) | 953 (9.79) |  |
| Others | 1555 (10.80) | 826 (17.71) | 729 (7.49) |  |
| Duration of surgery, n (%) |  |  |  | <0.001 |
| <60 min | 2069 (14.37) | 331 (7.10) | 1738 (17.85) |  |
| ≥60 min and <150 min | 7566 (52.54) | 2634 (56.46) | 4932 (50.66) |  |
| ≥150 min | 4766 (33.09) | 1700 (36.44) | 3066 (31.49) |  |
| Length of postoperative hospital stay, days | 6 (3, 9) | 10 (8, 14) | 4 (3, 6) | <0.001 |
| ICU admission, n (%) | 279 (1.94) | 221 (4.74) | 58 (0.60) | <0.001 |
| Stage of AKI, n (%) |  |  |  | <0.001 |
| Stage 1 | 210 (1.46) | 190 (4.07) | 20 (0.21) |  |
| Stage 2 | 31 (0.22) | 27 (0.58) | 4 (0.04) |  |
| Stage 3 | 18 (0.12) | 15 (0.32) | 3 (0.03) |  |
| TyG | 8.60 (8.22, 9.02) | 8.63 (8.25, 9.06) | 8.56 (8.19, 8.97) | <0.001 |
| TyG-BMI | 207.9 (181.9, 234.6) | 209.2 (182.6, 236.6) | 205.7 (181.3, 232.2) | <0.001 |
| NPAR | 14.64 (12.79, 17.10) | 15.58 (13.34, 19.09) | 14.33 (12.60, 16.38) | <0.001 |

ACEI/ARB:angiotensin converting enzyme inhibitor/angiotensin II receptor blockers; AKI: acute kidney injury; ASA: American Society of Anesthesiologists; BMI: body mass index; eGFR: estimated glomerular filtration rate; HDL-C: high-density lipoprotein cholesterol; ICU: intensive care unit; NPAR: neutrophil percentage to albumin ratio; TyG: triglyceride-glucose; TyG-BMI: triglyceride glucose-body mass index; UA: uric acid; WBC: white blood cell.

**Table S2** The number and percentage of patients with daily creatinine measurements after operation

| Time | Number of patients receiving creatinine determination | Percentage |
| --- | --- | --- |
| Preoperative | 4665 | 100% |
| Postoperative 1 day | 2057 | 44.09% |
| Postoperative 2 day | 1556 | 33.35% |
| Postoperative 3 day | 1503 | 32.22% |
| Postoperative 4 day | 1003 | 21.50% |
| Postoperative 5 day | 709 | 15.20% |
| Postoperative 6 day | 515 | 11.04% |
| Postoperative 7 day | 199 | 4.26% |

**Table S3** Predictive ability of TyG, TyG-BMI, NPAR, TyG & NPAR, TyG-BMI & NPAR for AKI

|  | **AUC (95% CI)** | **Sensitivity** | **Specificity** | ***P*-Value for AUC** | **Cutoff value** |
| --- | --- | --- | --- | --- | --- |
| TyG | 0.591 (0.552 - 0.630)^a^ | 0.560 | 0.592 | <0.001 | 8.757 |
| TyG-BMI | 0.559 (0.519 - 0.599)^a^ | 0.392 | 0.725 | 0.003 | 232.754 |
| NPAR | 0.655 (0.618 - 0.693)^b^ | 0.685 | 0.565 | <0.001 | 16.15 |
| TyG combined with NPAR | 0.670 (0.634 - 0.707)^b, c^ | 0.519 | 0.703 | <0.001 | – |
| TyG-BMI combined with NPAR | 0.683 (0.648 - 0.718)^c, d^ | 0.581 | 0.690 | <0.001 | – |

AKI: acute kidney injury; AUC: area under curve; CI: confidence interval; TyG: triglyceride-glucose; TyG-BMI: triglyceride glucose-body mass index; NPAR: neutrophil percentage to albumin ratio. ^a, b, c, d^ The same superscript letters indicate a non-significant difference of AUC.

**Table S4** Additive interaction between TyG and NPAR on AKI risk

| **Interactive indices** | **Interactive effects (95% CI)** | | |
| --- | --- | --- | --- |
|  | **Model 1** | **Model 2** | **Model 3** |
| Additive interaction |  |  |  |
| RERI | 1.785 (0.254, 3.316) | 1.294 (0.134, 2.364) | 0.653 (-0.381, 1.624) |
| AP | 0.328 (0.097, 0.559) | 0.322 (0.070, 0.574) | 0.232 (-0.090, 0.554) |
| SI | 1.670 (1.044, 2.672) | 1.768 (0.960, 3.253) | 1.562 (0.710, 3.437) |

AKI: acute kidney injury; AP: proportion attributable to interaction; CI: confidence interval; NPAR: neutrophil percentage to albumin ratio; RERI: relative excess risk due to interaction; SI: synergy index; TyG: triglyceride-glucose.

Model 1: unadjusted.

Model 2: adjusted for age, gender, smoking, drinking.

Model 3: further adjusted for diabetes mellitus, hypertension, hyperlipemia, anemia, diuretics, HDL-C, UA, sodium, potassium, calcium, eGFR, emergency, surgery type, anesthesia method, duration of surgery based on Model 2.

**Table S5** Additive interaction between TyG-BMI and NPAR on AKI risk

| **Interactive indices** | **Interactive effects (95% CI)** | | |
| --- | --- | --- | --- |
|  | **Model 1** | **Model 2** | **Model 3** |
| Additive interaction |  |  |  |
| RERI | 0.664 (-1.491, 2.819) | 0.523 (-1.191, 2.238) | 0.092 (-1.271, 1.456) |
| AP | 0.106 (-0.216, 0.428) | 0.108 (-0.226, 0.442) | 0.028 (-0.380, 0.436) |
| SI | 1.144 (0.742, 1.765) | 1.157 (0.715, 1.872) | 1.042 (0.568, 1.911) |

AKI: acute kidney injury; AP: proportion attributable to interaction; CI: confidence interval; NPAR: neutrophil percentage to albumin ratio; RERI: relative excess risk due to interaction; SI: synergy index; TyG-BMI: triglyceride glucose-body mass index.

Model 1: unadjusted.

Model 2: adjusted for age, gender, smoking, drinking.

Model 3: further adjusted for diabetes mellitus, hypertension, hyperlipemia, anemia, diuretics, HDL-C, UA, sodium, potassium, calcium, eGFR, emergency, surgery type, anesthesia method, duration of surgery based on Model 2.

**Table S6** Sensitivity analysis for the association of TyG, TyG-BMI, and NPAR with AKI after excluding spinal, osteotomy or deformity correction surgery (remaining patients, n = 3,510)

|  | **Model 1** | ***P* value** |  | **Model 2** | ***P* value** |  | **Model 3** | ***P* value** |
| --- | --- | --- | --- | --- | --- | --- | --- | --- |
|  | **OR (95% CI)** |  |  | **OR (95% CI)** |  |  | **OR (95% CI)** |  |
| TyG |  |  |  |  |  |  |  |  |
| Q1 | Reference |  |  | Reference |  |  | Reference |  |
| Q2 | 1.297 (0.809, 2.079) | 0.281 |  | 1.197 (0.742, 1.931) | 0.462 |  | 1.042 (0.640, 1.696) | 0.869 |
| Q3 | 1.803 (1.156, 2.814) | 0.009 |  | 1.581 (1.007, 2.482) | 0.047 |  | 1.408 (0.889, 2.228) | 0.144 |
| Q4 | 2.255 (1.467, 3.467) | <0.001 |  | 2.059 (1.331, 3.187) | 0.001 |  | 1.516 (0.952, 2.413) | 0.080 |
| *P* for trend | 1.781 (1.348, 2.351) | <0.001 |  | 1.694 (1.272, 2.254) | <0.001 |  | 1.169 (1.009, 1.353) | 0.037 |
| Per SD | 1.371 (1.202, 1.563) | <0.001 |  | 1.333 (1.162, 1.529) | <0.001 |  | 1.233 (1.056, 1.440) | 0.008 |
| TyG-BMI |  |  |  |  |  |  |  |  |
| Q1 | Reference |  |  | Reference |  |  | Reference |  |
| Q2 | 0.931 (0.605, 1.433) | 0.746 |  | 0.864 (0.557, 1.338) | 0.512 |  | 0.869 (0.549, 1.377) | 0.550 |
| Q3 | 0.954 (0.622, 1.465) | 0.831 |  | 0.871 (0.564, 1.346) | 0.535 |  | 0.785 (0.491, 1.255) | 0.312 |
| Q4 | 1.554 (1.053, 2.293) | 0.026 |  | 1.566 (1.050, 2.335) | 0.028 |  | 1.393 (0.907, 2.140) | 0.130 |
| *P* for trend | 1.005 (1.001, 1.009) | 0.016 |  | 1.005 (1.001, 1.010) | 0.016 |  | 1.118 (0.970, 1.290) | 0.124 |
| Per SD | 1.243 (1.083, 1.426) | 0.002 |  | 1.260 (1.092, 1.454) | 0.002 |  | 1.172 (1.010, 1.359) | 0.036 |
| NPAR |  |  |  |  |  |  |  |  |
| Q1 | Reference |  |  | Reference |  |  | Reference |  |
| Q2 | 1.001 (0.598, 1.676) | 0.996 |  | 0.907 (0.541, 1.523) | 0.712 |  | 1.156 (0.669, 1.997) | 0.604 |
| Q3 | 1.818 (1.150, 2.874) | 0.011 |  | 1.499 (0.941, 2.387) | 0.088 |  | 1.518 (0.903, 2.552) | 0.115 |
| Q4 | 3.030 (1.976, 4.645) | <0.001 |  | 2.001 (1.278, 3.134) | 0.002 |  | 1.809 (1.039, 3.147) | 0.036 |
| *P* for trend | 1.125 (1.084, 1.167) | <0.001 |  | 1.080 (1.039, 1.123) | <0.001 |  | 1.229 (1.033, 1.463) | 0.020 |
| Per SD | 1.479 (1.317, 1.662) | <0.001 |  | 1.317 (1.157, 1.499) | <0.001 |  | 1.249 (1.052, 1.483) | 0.011 |

AKI: acute kidney injury; CI: confidence interval; NPAR: neutrophil percentage to albumin ratio; OR: odds ratio; SD: standard deviation; TyG: triglyceride-glucose; TyG-BMI: triglyceride glucose-body mass index.

Model 1: unadjusted.

Model 2: adjusted for age, gender, smoking, drinking.

Model 3: further adjusted for diabetes mellitus, hypertension, hyperlipemia, anemia, diuretics, HDL-C, UA, sodium, potassium, calcium, eGFR, emergency, surgery type, anesthesia method, duration of surgery based on Model 2.

**Table S7** Sensitivity analysis for the association of TyG/TyG-BMI combined with NPAR with AKI after excluding spinal, osteotomy or deformity correction surgery (remaining patients, n = 3,510)

|  | **Model 1** | ***P* value** |  | **Model 2** | ***P* value** |  | **Model 3** | ***P* value** |
| --- | --- | --- | --- | --- | --- | --- | --- | --- |
|  | **OR (95% CI)** |  |  | **OR (95% CI)** |  |  | **OR (95% CI)** |  |
| TyG combined with NPAR |  |  |  |  |  |  |  |  |
| TyG low level & NPAR low level | Reference |  |  | Reference |  |  | Reference |  |
| TyG high level & NPAR low level | 3.066 (1.761, 5.337) | <0.001 |  | 2.616 (1.499, 4.567) | <0.001 |  | 1.566 (0.918, 2.672) | 0.099 |
| TyG low level & NPAR high level | 3.861 (2.262, 6.590) | <0.001 |  | 2.753 (1.592, 4.763) | <0.001 |  | 1.854 (1.107, 3.105) | 0.019 |
| TyG high level & NPAR high level | 5.976 (3.540, 10.087) | <0.001 |  | 4.201 (2.464, 7.162) | <0.001 |  | 3.108 (1.833, 5.268) | <0.001 |
| TyG-BMI combined with NPAR |  |  |  |  |  |  |  |  |
| TyG-BMI low level & NPAR low level | Reference |  |  | Reference |  |  | Reference |  |
| TyG-BMI high level & NPAR low level | 2.733 (1.643, 4.545) | <0.001 |  | 2.497 (1.496, 4.167) | <0.001 |  | 1.934 (1.137, 3.291) | 0.015 |
| TyG-BMI low level & NPAR high level | 3.331 (2.150, 5.158) | <0.001 |  | 2.376 (1.514, 3.727) | <0.001 |  | 2.042 (1.254, 3.325) | 0.004 |
| TyG-BMI high level & NPAR high level | 5.991 (3.662, 9.802) | <0.001 |  | 4.719 (2.862, 7.782) | <0.001 |  | 3.666 (2.149, 6.253) | <0.001 |

AKI: acute kidney injury; CI: confidence interval; NPAR: neutrophil percentage to albumin ratio; OR: odds ratio; TyG: triglyceride-glucose; TyG-BMI: triglyceride glucose-body mass index.

Model 1: unadjusted.

Model 2: adjusted for age, gender, smoking, drinking.

Model 3: further adjusted for diabetes mellitus, hypertension, hyperlipemia, anemia, diuretics, HDL-C, UA, sodium, potassium, calcium, eGFR, emergency, surgery type, anesthesia method, duration of surgery based on Model 2.

**Table S8** Sensitivity analysis for the association of TyG, TyG-BMI, and NPAR with AKI after excluding patients with diabetes (remaining patients, n = 4,111)

|  | **Model 1** | ***P* value** |  | **Model 2** | ***P* value** |  | **Model 3** | ***P* value** |
| --- | --- | --- | --- | --- | --- | --- | --- | --- |
|  | **OR (95% CI)** |  |  | **OR (95% CI)** |  |  | **OR (95% CI)** |  |
| TyG |  |  |  |  |  |  |  |  |
| Q1 | Reference |  |  | Reference |  |  | Reference |  |
| Q2 | 1.469 (0.908, 2.377) | 0.117 |  | 1.356 (0.833, 2.209) | 0.221 |  | 1.024 (0.632, 1.659) | 0.924 |
| Q3 | 1.798 (1.130, 2.861) | 0.013 |  | 1.613 (1.007, 2.584) | 0.047 |  | 1.450 (0.914, 2.301) | 0.114 |
| Q4 | 1.798 (1.130, 2.861) | 0.013 |  | 1.699 (1.060, 2.723) | 0.028 |  | 1.469 (0.911, 2.370) | 0.115 |
| *P* for trend | 1.476 (1.089, 2.001) | 0.012 |  | 1.434 (1.048, 1.962) | 0.024 |  | 1.165 (1.003, 1.353) | 0.046 |
| Per SD | 1.277 (1.110, 1.471) | <0.001 |  | 1.252 (1.081, 1.450) | 0.003 |  | 1.251 (1.062, 1.473) | 0.007 |
| TyG-BMI |  |  |  |  |  |  |  |  |
| Q1 | Reference |  |  | Reference |  |  | Reference |  |
| Q2 | 0.842 (0.525, 1.350) | 0.475 |  | 0.794 (0.492, 1.282) | 0.345 |  | 0.968 (0.593, 1.578) | 0.895 |
| Q3 | 1.080 (0.692, 1.685) | 0.734 |  | 0.983 (0.626, 1.544) | 0.940 |  | 1.229 (0.764, 1.976) | 0.395 |
| Q4 | 1.544 (1.021, 2.336) | 0.040 |  | 1.573 (1.027, 2.410) | 0.037 |  | 1.861 (1.175, 2.948) | 0.008 |
| *P* for trend | 1.006 (1.001, 1.010) | 0.015 |  | 1.006 (1.001, 1.011) | 0.014 |  | 1.246 (1.072, 1.448) | 0.004 |
| Per SD | 1.236 (1.069, 1.430) | 0.004 |  | 1.264 (1.084, 1.473) | 0.003 |  | 1.296 (1.106, 1.519) | 0.001 |
| NPAR |  |  |  |  |  |  |  |  |
| Q1 | Reference |  |  | Reference |  |  | Reference |  |
| Q2 | 1.488 (0.826, 2.683) | 0.186 |  | 1.316 (0.728, 2.377) | 0.364 |  | 1.201 (0.667, 2.163) | 0.542 |
| Q3 | 2.150 (1.237, 3.738) | 0.007 |  | 1.728 (0.987, 3.023) | 0.055 |  | 1.488 (0.850, 2.608) | 0.165 |
| Q4 | 4.848 (2.927, 8.031) | <0.001 |  | 3.085 (1.823, 5.219) | <0.001 |  | 2.179 (1.212, 3.918) | 0.009 |
| *P* for trend | 1.171 (1.124, 1.221) | <0.001 |  | 1.120 (1.072, 1.170) | <0.001 |  | 1.302 (1.081, 1.569) | 0.005 |
| Per SD | 1.685 (1.505, 1.887) | <0.001 |  | 1.509 (1.332, 1.710) | <0.001 |  | 1.410 (1.183, 1.680) | <0.001 |

AKI: acute kidney injury; CI: confidence interval; NPAR: neutrophil percentage to albumin ratio; OR: odds ratio; SD: standard deviation; TyG: triglyceride-glucose; TyG-BMI: triglyceride glucose-body mass index.

Model 1: unadjusted.

Model 2: adjusted for age, gender, smoking, drinking.

Model 3: further adjusted for diabetes mellitus, hypertension, hyperlipemia, anemia, diuretics, HDL-C, UA, sodium, potassium, calcium, eGFR, emergency, surgery type, anesthesia method, duration of surgery based on Model 2.

**Table S9** Sensitivity analysis for the association of TyG/TyG-BMI combined with NPAR with AKI after excluding patients with diabetes (remaining patients, n = 4,111)

|  | **Model 1** | ***P* value** |  | **Model 2** | ***P* value** |  | **Model 3** | ***P* value** |
| --- | --- | --- | --- | --- | --- | --- | --- | --- |
|  | **OR (95% CI)** |  |  | **OR (95% CI)** |  |  | **OR (95% CI)** |  |
| TyG combined with NPAR |  |  |  |  |  |  |  |  |
| TyG low level & NPAR low level | Reference |  |  | Reference |  |  | Reference |  |
| TyG high level & NPAR low level | 1.715 (1.096, 2.683) | 0.018 |  | 1.519 (0.968, 2.382) | 0.069 |  | 1.606 (0.916, 2.815) | 0.098 |
| TyG low level & NPAR high level | 3.320 (2.154, 5.117) | <0.001 |  | 2.121 (1.344, 3.346) | 0.001 |  | 1.973 (1.168, 3.332) | 0.011 |
| TyG high level & NPAR high level | 5.577 (3.570, 8.711) | <0.001 |  | 3.964 (2.504, 6.275) | <0.001 |  | 3.251 (1.886, 5.603) | <0.001 |
| TyG-BMI combined with NPAR |  |  |  |  |  |  |  |  |
| TyG-BMI low level & NPAR low level | Reference |  |  | Reference |  |  | Reference |  |
| TyG-BMI high level & NPAR low level | 2.317 (1.479, 3.629) | <0.001 |  | 2.173 (1.383, 3.416) | <0.001 |  | 2.494 (1.418, 4.387) | 0.002 |
| TyG-BMI low level & NPAR high level | 3.856 (2.590, 5.739) | <0.001 |  | 2.555 (1.683, 3.880) | <0.001 |  | 2.346 (1.406, 3.914) | 0.001 |
| TyG-BMI high level & NPAR high level | 6.913 (4.186, 11.417) | <0.001 |  | 5.264 (3.148, 8.801) | <0.001 |  | 4.137 (2.347, 7.290) | <0.001 |

AKI: acute kidney injury; CI: confidence interval; NPAR: neutrophil percentage to albumin ratio; OR: odds ratio; TyG: triglyceride-glucose; TyG-BMI: triglyceride glucose-body mass index.

Model 1: unadjusted.

Model 2: adjusted for age, gender, smoking, drinking.

Model 3: further adjusted for diabetes mellitus, hypertension, hyperlipemia, anemia, diuretics, HDL-C, UA, sodium, potassium, calcium, eGFR, emergency, surgery type, anesthesia method, duration of surgery based on Model 2.

**Table S10** Sensitivity analysis for the association of TyG, TyG-BMI, and NPAR with AKI after excluding patients with hyperlipemia (remaining patients, n = 4,616)

|  | **Model 1** | ***P* value** |  | **Model 2** | ***P* value** |  | **Model 3** | ***P* value** |
| --- | --- | --- | --- | --- | --- | --- | --- | --- |
|  | **OR (95% CI)** |  |  | **OR (95% CI)** |  |  | **OR (95% CI)** |  |
| TyG |  |  |  |  |  |  |  |  |
| Q1 | Reference |  |  | Reference |  |  | Reference |  |
| Q2 | 1.156 (0.751, 1.781) | 0.510 |  | 1.059 (0.684, 1.640) | 0.796 |  | 1.025 (0.653, 1.609) | 0.915 |
| Q3 | 1.581 (1.053, 2.373) | 0.027 |  | 1.375 (0.911, 2.075) | 0.130 |  | 1.374 (0.895, 2.108) | 0.146 |
| Q4 | 2.047 (1.386, 3.021) | <.001 |  | 1.853 (1.248, 2.751) | 0.002 |  | 1.516 (0.989, 2.324) | 0.566 |
| *P* for trend | 1.687 (1.309, 2.176) | <0.001 |  | 1.597 (1.230, 2.074) | <0.001 |  | 1.169 (1.022, 1.338) | 0.023 |
| Per SD | 1.370 (1.213, 1.548) | <0.001 |  | 1.329 (1.171, 1.507) | <0.001 |  | 1.244 (1.081, 1.431) | 0.002 |
| TyG-BMI |  |  |  |  |  |  |  |  |
| Q1 | Reference |  |  | Reference |  |  | Reference |  |
| Q2 | 1.021 (0.685, 1.521) | 0.919 |  | 0.972 (0.649, 1.457) | 0.892 |  | 1.047 (0.685, 1.602) | 0.832 |
| Q3 | 0.937 (0.624, 1.408) | 0.756 |  | 0.866 (0.574, 1.308) | 0.494 |  | 0.968 (0.627, 1.496) | 0.885 |
| Q4 | 1.623 (1.128, 2.335) | 0.009 |  | 1.643 (1.132, 2.387) | 0.009 |  | 1.643 (1.097, 2.460) | 0.016 |
| *P* for trend | 1.005 (1.001, 1.009) | 0.008 |  | 1.006 (1.002, 1.010) | 0.007 |  | 1.170 (1.025, 1.335) | 0.020 |
| Per SD | 1.230 (1.083, 1.397) | 0.001 |  | 1.248 (1.093, 1.425) | 0.001 |  | 1.218 (1.061, 1.398) | 0.005 |
| NPAR |  |  |  |  |  |  |  |  |
| Q1 | Reference |  |  | Reference |  |  | Reference |  |
| Q2 | 1.241 (0.761, 2.023) | 0.386 |  | 1.109 (0.679, 1.812) | 0.680 |  | 1.013 (0.614, 1.671) | 0.960 |
| Q3 | 1.875 (1.193, 2.948) | 0.006 |  | 1.521 (0.961, 2.406) | 0.073 |  | 1.278 (0.795, 2.054) | 0.312 |
| Q4 | 3.750 (2.478, 5.676) | <0.001 |  | 2.474 (1.604, 3.815) | <0.001 |  | 1.598 (0.971, 2.630) | 0.065 |
| *P* for trend | 1.142 (1.104, 1.182) | <0.001 |  | 1.098 (1.058, 1.138) | <0.001 |  | 1.187 (1.011, 1.393) | 0.036 |
| Per SD | 1.577 (1.425, 1.746) | <0.001 |  | 1.419 (1.269, 1.587) | <0.001 |  | 1.209 (1.041, 1.403) | 0.013 |

AKI: acute kidney injury; CI: confidence interval; NPAR: neutrophil percentage to albumin ratio; OR: odds ratio; SD: standard deviation; TyG: triglyceride-glucose; TyG-BMI: triglyceride glucose-body mass index.

Model 1: unadjusted.

Model 2: adjusted for age, gender, smoking, drinking.

Model 3: further adjusted for diabetes mellitus, hypertension, hyperlipemia, anemia, diuretics, HDL-C, UA, sodium, potassium, calcium, eGFR, emergency, surgery type, anesthesia method, duration of surgery based on Model 2.

**Table S11** Sensitivity analysis for the association of TyG/TyG-BMI combined with NPAR with AKI after excluding patients with hyperlipemia (remaining patients, n = 4,616)

|  | **Model 1** | ***P* value** |  | **Model 2** | ***P* value** |  | **Model 3** | ***P* value** |
| --- | --- | --- | --- | --- | --- | --- | --- | --- |
|  | **OR (95% CI)** |  |  | **OR (95% CI)** |  |  | **OR (95% CI)** |  |
| TyG combined with NPAR |  |  |  |  |  |  |  |  |
| TyG low level & NPAR low level | Reference |  |  | Reference |  |  | Reference |  |
| TyG high level & NPAR low level | 1.777 (1.104, 2.859) | 0.018 |  | 1.563 (0.969, 2.520) | 0.067 |  | 1.452 (0.888, 2.374) | 0.137 |
| TyG low level & NPAR high level | 2.770 (1.802, 4.257) | <0.001 |  | 1.977 (1.269, 3.079) | 0.003 |  | 1.639 (1.022, 2.630) | 0.041 |
| TyG high level & NPAR high level | 5.365 (3.525, 8.166) | <0.001 |  | 3.819 (2.485, 5.870) | <0.001 |  | 2.814 (1.744, 4.542) | <0.001 |
| TyG-BMI combined with NPAR |  |  |  |  |  |  |  |  |
| TyG-BMI low level & NPAR low level | Reference |  |  | Reference |  |  | Reference |  |
| TyG-BMI high level & NPAR low level | 2.647 (1.646, 4.256) | <0.001 |  | 2.452 (1.521, 3.953) | <0.001 |  | 2.075 (1.267, 3.398) | 0.004 |
| TyG-BMI low level & NPAR high level | 3.765 (2.521, 5.625) | <0.001 |  | 2.716 (1.797, 4.105) | <0.001 |  | 2.039 (1.299, 3.201) | 0.002 |
| TyG-BMI high level & NPAR high level | 6.310 (3.998, 9.958) | <0.001 |  | 4.898 (3.080, 7.787) | <0.001 |  | 3.322 (2.020, 5.462) | <0.001 |

AKI: acute kidney injury; CI: confidence interval; NPAR: neutrophil percentage to albumin ratio; OR: odds ratio; TyG: triglyceride-glucose; TyG-BMI: triglyceride glucose-body mass index.

Model 1: unadjusted.

Model 2: adjusted for age, gender, smoking, drinking.

Model 3: further adjusted for diabetes mellitus, hypertension, hyperlipemia, anemia, diuretics, HDL-C, UA, sodium, potassium, calcium, eGFR, emergency, surgery type, anesthesia method, duration of surgery based on Model 2.

**Table S12** Sensitivity analysis for the association of TyG, TyG-BMI, and NPAR with AKI after excluding patients with emergency surgery (remaining patients, n = 4,526)

|  | **Model 1** | ***P* value** |  | **Model 2** | ***P* value** |  | **Model 3** | ***P* value** |
| --- | --- | --- | --- | --- | --- | --- | --- | --- |
|  | **OR (95% CI)** |  |  | **OR (95% CI)** |  |  | **OR (95% CI)** |  |
| TyG |  |  |  |  |  |  |  |  |
| Q1 | Reference |  |  | Reference |  |  | Reference |  |
| Q2 | 1.291 (0.828, 2.012) | 0.260 |  | 1.186 (0.757, 1.858) | 0.457 |  | 1.075 (0.677, 1.706) | 0.759 |
| Q3 | 1.766 (1.161, 2.685) | 0.008 |  | 1.533 (1.003, 2.344) | 0.048 |  | 1.554 (1.008, 2.394) | 0.046 |
| Q4 | 2.129 (1.417, 3.199) | <0.001 |  | 1.939 (1.284, 2.930) | 0.002 |  | 1.688 (1.100, 2.589) | 0.017 |
| *P* for trend | 1.701 (1.309, 2.210) | <0.001 |  | 1.615 (1.234, 2.114) | <0.001 |  | 1.214 (1.062, 1.387) | 0.005 |
| Per SD | 1.345 (1.187, 1.524) | <0.001 |  | 1.303 (1.145, 1.482) | <0.001 |  | 1.264 (1.101, 1.451) | <0.001 |
| TyG-BMI |  |  |  |  |  |  |  |  |
| Q1 | Reference |  |  | Reference |  |  | Reference |  |
| Q2 | 0.957 (0.633, 1.447) | 0.837 |  | 0.903 (0.594, 1.373) | 0.633 |  | 0.986 (0.636, 1.529) | 0.951 |
| Q3 | 0.979 (0.649, 1.477) | 0.920 |  | 0.900 (0.593, 1.364) | 0.619 |  | 0.968 (0.621, 1.508) | 0.885 |
| Q4 | 1.648 (1.138, 2.387) | 0.008 |  | 1.648 (1.127, 2.409) | 0.010 |  | 1.652 (1.098, 2.484) | 0.016 |
| *P* for trend | 1.006 (1.002, 1.010) | 0.004 |  | 1.006 (1.002, 1.010) | 0.005 |  | 1.184 (1.035, 1.353) | 0.014 |
| Per SD | 1.245 (1.093, 1.417) | <0.001 |  | 1.258 (1.099, 1.440) | <0.001 |  | 1.230 (1.070, 1.415) | 0.004 |
| NPAR |  |  |  |  |  |  |  |  |
| Q1 | Reference |  |  | Reference |  |  | Reference |  |
| Q2 | 1.242 (0.762, 2.025) | 0.384 |  | 1.107 (0.678, 1.810) | 0.684 |  | 1.060 (0.646, 1.740) | 0.818 |
| Q3 | 1.770 (1.121, 2.796) | 0.014 |  | 1.438 (0.905, 2.286) | 0.124 |  | 1.263 (0.785, 2.031) | 0.335 |
| Q4 | 3.520 (2.320, 5.343) | <0.001 |  | 2.309 (1.491, 3.573) | <0.001 |  | 1.536 (0.926, 2.548) | 0.096 |
| *P* for trend | 1.141 (1.100, 1.183) | <0.001 |  | 1.094 (1.052, 1.137) | <0.001 |  | 1.162 (0.989, 1.367) | 0.068 |
| Per SD | 1.535 (1.380, 1.707) | <0.001 |  | 1.361 (1.210, 1.531) | <0.001 |  | 1.176 (1.002, 1.381) | 0.048 |

AKI: acute kidney injury; CI: confidence interval; NPAR: neutrophil percentage to albumin ratio; OR: odds ratio; SD: standard deviation; TyG: triglyceride-glucose; TyG-BMI: triglyceride glucose-body mass index.

Model 1: unadjusted.

Model 2: adjusted for age, gender, smoking, drinking.

Model 3: further adjusted for diabetes mellitus, hypertension, hyperlipemia, anemia, diuretics, HDL-C, UA, sodium, potassium, calcium, eGFR, emergency, surgery type, anesthesia method, duration of surgery based on Model 2.

**Table S13** Sensitivity analysis for the association of TyG/TyG-BMI combined with NPAR with AKI after excluding patients with emergency surgery (remaining patients, n = 4,526)

|  | **Model 1** | ***P* value** |  | **Model 2** | ***P* value** |  | **Model 3** | ***P* value** |
| --- | --- | --- | --- | --- | --- | --- | --- | --- |
|  | **OR (95% CI)** |  |  | **OR (95% CI)** |  |  | **OR (95% CI)** |  |
| TyG combined with NPAR |  |  |  |  |  |  |  |  |
| TyG low level & NPAR low level | Reference |  |  | Reference |  |  | Reference |  |
| TyG high level & NPAR low level | 2.483 (1.506, 4.093) | <0.001 |  | 2.124 (1.285, 3.510) | 0.003 |  | 1.560 (0.960, 2.534) | 0.073 |
| TyG low level & NPAR high level | 3.563 (2.195, 5.783) | <0.001 |  | 2.496 (1.517, 4.106) | <0.001 |  | 1.581 (0.980, 2.552) | 0.061 |
| TyG high level & NPAR high level | 5.735 (3.582, 9.181) | <0.001 |  | 3.975 (2.458, 6.429) | <0.001 |  | 2.884 (1.785, 4.658) | <0.001 |
| TyG-BMI combined with NPAR |  |  |  |  |  |  |  |  |
| TyG-BMI low level & NPAR low level | Reference |  |  | Reference |  |  | Reference |  |
| TyG-BMI high level & NPAR low level | 2.746 (1.717, 4.394) | <0.001 |  | 2.519 (1.571, 4.041) | <0.001 |  | 2.204 (1.355, 3.584) | 0.002 |
| TyG-BMI low level & NPAR high level | 3.620 (2.414, 5.429) | <0.001 |  | 2.579 (1.698, 3.917) | <0.001 |  | 2.001 (1.270, 3.153) | 0.003 |
| TyG-BMI high level & NPAR high level | 6.088 (3.835, 9.663) | <0.001 |  | 4.676 (2.923, 7.481) | <0.001 |  | 3.303 (1.998, 5.461) | <0.001 |

AKI: acute kidney injury; CI: confidence interval; NPAR: neutrophil percentage to albumin ratio; OR: odds ratio; TyG: triglyceride-glucose; TyG-BMI: triglyceride glucose-body mass index.

Model 1: unadjusted.

Model 2: adjusted for age, gender, smoking, drinking.

Model 3: further adjusted for diabetes mellitus, hypertension, hyperlipemia, anemia, diuretics, HDL-C, UA, sodium, potassium, calcium, eGFR, emergency, surgery type, anesthesia method, duration of surgery based on Model 2.

**Table S14** Sensitivity analysis for the association of TyG, TyG-BMI, and NPAR with AKI after excluding patients with emergency surgery or neutrophil percentage > 75% (remaining patients, n = 3,751)

|  | **Model 1** | ***P* value** | **Model 2** | ***P* value** | **Model 3** | ***P* value** |
| --- | --- | --- | --- | --- | --- | --- |
|  | **OR (95% CI)** |  | **OR (95% CI)** |  | **OR (95% CI)** |  |
| TyG |  |  |  |  |  |  |
| Q1 | Reference |  | Reference |  | Reference |  |
| Q2 | 1.285 (0.773, 2.137) | 0.333 | 1.131 (0.675, 1.895) | 0.639 | 1.131 (0.664, 1.926) | 0.650 |
| Q3 | 2.064 (1.296, 3.288) | 0.002 | 1.659 (1.033, 2.667) | 0.036 | 1.794 (1.097, 2.932) | 0.020 |
| Q4 | 1.859 (1.149, 3.007) | 0.012 | 1.649 (1.011, 2.689) | 0.045 | 1.578 (0.944, 2.639) | 0.082 |
| *P* for trend | 1.252 (1.084, 1.445) | 0.002 | 1.205 (1.037, 1.400) | 0.015 | 1.196 (1.022, 1.399) | 0.026 |
| Per SD | 1.289 (1.110, 1.496) | <0.001 | 1.234 (1.053, 1.445) | 0.009 | 1.219 (1.032, 1.440) | 0.020 |
| TyG-BMI |  |  |  |  |  |  |
| Q1 | Reference |  | Reference |  | Reference |  |
| Q2 | 1.101 (0.660, 1.836) | 0.712 | 0.998 (0.593, 1.679) | 0.994 | 1.018 (0.593, 1.748) | 0.949 |
| Q3 | 1.122 (0.678, 1.858) | 0.654 | 0.948 (0.568, 1.582) | 0.838 | 0.995 (0.581, 1.704) | 0.984 |
| Q4 | 2.136 (1.367, 3.338) | <0.001 | 1.939 (1.224, 3.070) | 0.005 | 1.845 (1.126, 3.024) | 0.015 |
| *P* for trend | 1.300 (1.124, 1.503) | <0.001 | 1.264 (1.086, 1.470) | 0.002 | 1.241 (1.058, 1.457) | 0.008 |
| Per SD | 1.337 (1.151, 1.553) | <0.001 | 1.309 (1.118, 1.532) | <0.001 | 1.260 (1.071, 1.484) | 0.006 |
| NPAR |  |  |  |  |  |  |
| Q1 | Reference |  | Reference |  | Reference |  |
| Q2 | 1.318 (0.813, 2.136) | 0.263 | 1.139 (0.700, 1.854) | 0.601 | 1.058 (0.643, 1.739) | 0.825 |
| Q3 | 1.978 (1.254, 3.120) | 0.003 | 1.466 (0.919, 2.338) | 0.108 | 1.275 (0.786, 2.068) | 0.324 |
| Q4 | 4.179 (2.558, 6.827) | <0.001 | 2.389 (1.421, 4.017) | 0.001 | 1.894 (1.051, 3.413) | 0.034 |
| *P* for trend | 1.600 (1.363, 1.878) | <0.001 | 1.330 (1.124, 1.573) | <0.001 | 1.221 (1.011, 1.473) | 0.038 |
| Per SD | 2.162 (1.732, 2.700) | <0.001 | 1.666 (1.308, 2.121) | <0.001 | 1.462 (1.102, 1.939) | 0.009 |

AKI: acute kidney injury; CI: confidence interval; NPAR: neutrophil percentage to albumin ratio; OR: odds ratio; SD: standard deviation; TyG: triglyceride-glucose; TyG-BMI: triglyceride glucose-body mass index.

Model 1: unadjusted.

Model 2: adjusted for age, gender, smoking, drinking.

Model 3: further adjusted for diabetes mellitus, hypertension, hyperlipemia, anemia, diuretics, HDL-C, UA, sodium, potassium, calcium, eGFR, emergency, surgery type, anesthesia method, duration of surgery based on Model 2.

**Table S15** Sensitivity analysis for the association of TyG/TyG-BMI combined with NPAR with AKI after excluding patients with emergency surgery or neutrophil percentage > 75% (remaining patients, n = 3,751)

|  | **Model 1** | ***P* value** |  | **Model 2** | ***P* value** |  | **Model 3** | ***P* value** |
| --- | --- | --- | --- | --- | --- | --- | --- | --- |
|  | **OR (95% CI)** |  |  | **OR (95% CI)** |  |  | **OR (95% CI)** |  |
| TyG combined with NPAR |  |  |  |  |  |  |  |  |
| TyG low level & NPAR low level | Reference |  |  | Reference |  |  | Reference |  |
| TyG high level & NPAR low level | 1.866 (1.165, 2.988) | 0.009 |  | 1.602 (0.997, 2.574) | 0.052 |  | 1.509 (0.926, 2.458) | 0.099 |
| TyG low level & NPAR high level | 2.775 (1.737, 4.431) | <0.001 |  | 1.879 (1.155, 3.055) | 0.011 |  | 1.610 (0.959, 2.702) | 0.072 |
| TyG high level & NPAR high level | 5.235 (3.262, 8.402) | <0.001 |  | 3.466 (2.135, 5.627) | <0.001 |  | 3.074 (1.831, 5.161) | <0.001 |
| TyG-BMI combined with NPAR |  |  |  |  |  |  |  |  |
| TyG-BMI low level & NPAR low level | Reference |  |  | Reference |  |  | Reference |  |
| TyG-BMI high level & NPAR low level | 2.750 (1.719, 4.400) | <0.001 |  | 2.482 (1.545, 3.988) | <0.001 |  | 2.192 (1.344, 3.577) | 0.002 |
| TyG-BMI low level & NPAR high level | 3.498 (2.251, 5.435) | <0.001 |  | 2.428 (1.539, 3.829) | <0.001 |  | 2.092 (1.284, 3.408) | 0.003 |
| TyG-BMI high level & NPAR high level | 6.23 (3.738, 10.384) | <0.001 |  | 4.320 (2.562, 7.286) | <0.001 |  | 3.386 (1.951, 5.875) | <0.001 |

AKI: acute kidney injury; CI: confidence interval; NPAR: neutrophil percentage to albumin ratio; OR: odds ratio; TyG: triglyceride-glucose; TyG-BMI: triglyceride glucose-body mass index.

Model 1: unadjusted.

Model 2: adjusted for age, gender, smoking, drinking.

Model 3: further adjusted for diabetes mellitus, hypertension, hyperlipemia, anemia, diuretics, HDL-C, UA, sodium, potassium, calcium, eGFR, emergency, surgery type, anesthesia method, duration of surgery based on Model 2.

**Table S16** Sensitivity analysis for the association of TyG, TyG-BMI, and NPAR with AKI after including patients with complete exposure indices in the exclusion population (n = 8,450)

|  | **Model 1** | ***P value*** |  | **Model 2** | ***P value*** |  | **Model 3** | ***P value*** |
| --- | --- | --- | --- | --- | --- | --- | --- | --- |
|  | **OR (95% CI)** |  |  | **OR (95% CI)** |  |  | **OR (95% CI)** |  |
| TyG |  |  |  |  |  |  |  |  |
| Q1 | Reference |  |  | Reference |  |  | Reference |  |
| Q2 | 1.369 (0.887, 2.114) | 0.157 |  | 1.240 (0.798, 1.924) | 0.339 |  | 1.106 (0.701, 1.744) | 0.665 |
| Q3 | 1.743 (1.151, 2.640) | 0.009 |  | 1.520 (0.997, 2.315) | 0.051 |  | 1.287 (0.829, 1.998) | 0.262 |
| Q4 | 2.418 (1.630, 3.588) | <0.001 |  | 2.149 (1.440, 3.208) | <0.001 |  | 1.410 (0.913, 2.177) | 0.122 |
| *P* for trend | 1.337 (1.185, 1.508) | <0.001 |  | 1.294 (1.143, 1.465) | <0.001 |  | 1.125 (0.983, 1.287) | 0.087 |
| Per SD | 1.444 (1.287, 1.621) | <0.001 |  | 1.393 (1.235, 1.571) | <0.001 |  | 1.207 (1.052, 1.383) | 0.007 |
| TyG-BMI |  |  |  |  |  |  |  |  |
| Q1 | Reference |  |  | Reference |  |  | Reference |  |
| Q2 | 0.897 (0.597, 1.349) | 0.602 |  | 0.859 (0.568, 1.299) | 0.470 |  | 0.845 (0.548, 1.303) | 0.446 |
| Q3 | 1.041 (0.702, 1.541) | 0.843 |  | 0.974 (0.653, 1.451) | 0.896 |  | 0.972 (0.635, 1.489) | 0.897 |
| Q4 | 1.729 (1.213, 2.466) | 0.003 |  | 1.757 (1.221, 2.529) | 0.002 |  | 1.561 (1.049, 2.323) | 0.028 |
| *P* for trend | 1.222 (1.086, 1.376) | <0.001 |  | 1.231 (1.089, 1.392) | <0.001 |  | 1.186 (1.040, 1.352) | 0.011 |
| Per SD | 1.282 (1.134, 1.449) | <0.001 |  | 1.300 (1.144, 1.476) | <0.001 |  | 1.212 (1.062, 1.383) | 0.004 |
| NPAR |  |  |  |  |  |  |  |  |
| Q1 | Reference |  |  | Reference |  |  | Reference |  |
| Q2 | 1.677 (0.973, 2.891) | 0.063 |  | 1.467 (0.849, 2.534) | 0.169 |  | 1.345 (0.771, 2.347) | 0.296 |
| Q3 | 2.118 (1.255, 3.574) | 0.005 |  | 1.689 (0.997, 2.860) | 0.051 |  | 1.414 (0.825, 2.425) | 0.207 |
| Q4 | 6.638 (4.172, 10.563) | <0.001 |  | 3.734 (2.303, 6.056) | <0.001 |  | 2.107 (1.237, 3.588) | 0.006 |
| *P* for trend | 1.972 (1.717, 2.265) | <0.001 |  | 1.584 (1.372, 1.829) | <0.001 |  | 1.266 (1.077, 1.489) | 0.004 |
| Per SD | 1.772 (1.626, 1.930) | <0.001 |  | 1.550 (1.409, 1.706) | <0.001 |  | 1.287 (1.128, 1.468) | <0.001 |

AKI: acute kidney injury; CI: confidence interval; NPAR: neutrophil percentage to albumin ratio; OR: odds ratio; SD: standard deviation; TyG: triglyceride-glucose; TyG-BMI: triglyceride glucose-body mass index.

Model 1: unadjusted.

Model 2: adjusted for age, gender, smoking, drinking.

Model 3: further adjusted for diabetes mellitus, hypertension, hyperlipemia, anemia, diuretics, HDL-C, UA, sodium, potassium, calcium, eGFR, emergency, surgery type, anesthesia method, duration of surgery based on Model 2.

**Table S17** Sensitivity analysis for the association of TyG/TyG-BMI combined with NPAR with AKI after including patients with complete exposure indices in the exclusion population (n = 8,450)

|  | **Model 1** | ***P* value** |  | **Model 2** | ***P* value** |  | **Model 3** | ***P* value** |
| --- | --- | --- | --- | --- | --- | --- | --- | --- |
|  | **OR (95% CI)** |  |  | **OR (95% CI)** |  |  | **OR (95% CI)** |  |
| TyG combined with NPAR |  |  |  |  |  |  |  |  |
| TyG low level & NPAR low level | Reference |  |  | Reference |  |  | Reference |  |
| TyG high level & NPAR low level | 2.054 (1.287, 3.277) | 0.003 |  | 1.789 (1.119, 2.859) | 0.015 |  | 1.467 (0.905, 2.377) | 0.120 |
| TyG low level & NPAR high level | 4.367 (2.852, 6.686) | <0.001 |  | 2.715 (1.746, 4.222) | <0.001 |  | 1.923 (1.202, 3.075) | 0.006 |
| TyG high level & NPAR high level | 9.112 (6.015, 13.803) | <0.001 |  | 5.649 (3.686, 8.655) | <0.001 |  | 3.021 (1.872, 4.874) | <0.001 |
| TyG-BMI combined with NPAR |  |  |  |  |  |  |  |  |
| TyG-BMI low level & NPAR low level | Reference |  |  | Reference |  |  | Reference |  |
| TyG-BMI high level & NPAR low level | 3.216 (2.018, 5.125) | <0.001 |  | 2.975 (1.863, 4.750) | <0.001 |  | 2.347 (1.450, 3.798) | <0.001 |
| TyG-BMI low level & NPAR high level | 6.166 (4.143, 9.175) | <0.001 |  | 3.892 (2.579, 5.873) | <0.001 |  | 2.449 (1.567, 3.828) | <0.001 |
| TyG-BMI high level & NPAR high level | 10.281 (6.542, 16.156) | <0.001 |  | 7.026 (4.43, 11.143) | <0.001 |  | 3.812 (2.320, 6.263) | <0.001 |

AKI: acute kidney injury; CI: confidence interval; NPAR: neutrophil percentage to albumin ratio; OR: odds ratio; TyG: triglyceride-glucose; TyG-BMI: triglyceride glucose-body mass index.

Model 1: unadjusted.

Model 2: adjusted for age, gender, smoking, drinking.

Model 3: further adjusted for diabetes mellitus, hypertension, hyperlipemia, anemia, diuretics, HDL-C, UA, sodium, potassium, calcium, eGFR, emergency, surgery type, anesthesia method, duration of surgery based on Model 2.

**Figure S1** Sensitivity analysis for RCS curve between TyG, TyG-BMI, and NPAR and the risk of AKI after excluding spinal, osteotomy or deformity correction surgery (remaining patients, n = 3,510). (A) TyG and AKI. (B) TyG-BMI and AKI. (C) NPAR and AKI. AKI: acute kidney injury; NPAR: neutrophil percentage to albumin ratio; RCS: restricted cubic spline; TyG: triglyceride-glucose; TyG-BMI: triglyceride glucose-body mass index. All analyses were adjusted for confounding factors including age, gender, smoking, drinking, diabetes mellitus, hypertension, hyperlipemia, anemia, diuretics, HDL-C, UA, sodium, potassium, calcium, eGFR, emergency, surgery type, anesthesia method, duration of surgery.

**Figure S2** Sensitivity analysis for RCS curve between TyG, TyG-BMI, and NPAR and the risk of AKI after excluding patients with diabetes (remaining patients, n = 4,111). (A) TyG and AKI. (B) TyG-BMI and AKI. (C) NPAR and AKI. AKI: acute kidney injury; NPAR: neutrophil percentage to albumin ratio; RCS: restricted cubic spline; TyG: triglyceride-glucose; TyG-BMI: triglyceride glucose-body mass index. All analyses were adjusted for confounding factors including age, gender, smoking, drinking, diabetes mellitus, hypertension, hyperlipemia, anemia, diuretics, HDL-C, UA, sodium, potassium, calcium, eGFR, emergency, surgery type, anesthesia method, duration of surgery.

**Figure S3** Sensitivity analysis for RCS curve between TyG, TyG-BMI, and NPAR and the risk of AKI after excluding patients with hyperlipemia (remaining patients, n = 4,616). (A) TyG and AKI. (B) TyG-BMI and AKI. (C) NPAR and AKI. AKI: acute kidney injury; NPAR: neutrophil percentage to albumin ratio; RCS: restricted cubic spline; TyG: triglyceride-glucose; TyG-BMI: triglyceride glucose-body mass index. All analyses were adjusted for confounding factors including age, gender, smoking, drinking, diabetes mellitus, hypertension, hyperlipemia, anemia, diuretics, HDL-C, UA, sodium, potassium, calcium, eGFR, emergency, surgery type, anesthesia method, duration of surgery.

**Figure S4** Sensitivity analysis for RCS curve between TyG, TyG-BMI, and NPAR and the risk of AKI after excluding patients with emergency surgery (remaining patients, n = 4,526). (A) TyG and AKI. (B) TyG-BMI and AKI. (C) NPAR and AKI. AKI: acute kidney injury; NPAR: neutrophil percentage to albumin ratio; RCS: restricted cubic spline; TyG: triglyceride-glucose; TyG-BMI: triglyceride glucose-body mass index. All analyses were adjusted for confounding factors including age, gender, smoking, drinking, diabetes mellitus, hypertension, hyperlipemia, anemia, diuretics, HDL-C, UA, sodium, potassium, calcium, eGFR, emergency, surgery type, anesthesia method, duration of surgery.

**Figure S5** Sensitivity analysis for RCS curve between TyG, TyG-BMI, and NPAR and the risk of AKI after excluding patients with emergency surgery or neutrophil percentage > 75% (remaining patients, n = 3,751). (A) TyG and AKI. (B) TyG-BMI and AKI. (C) NPAR and AKI. AKI: acute kidney injury; NPAR: neutrophil percentage to albumin ratio; RCS: restricted cubic spline; TyG: triglyceride-glucose; TyG-BMI: triglyceride glucose-body mass index. All analyses were adjusted for confounding factors including age, gender, smoking, drinking, diabetes mellitus, hypertension, hyperlipemia, anemia, diuretics, HDL-C, UA, sodium, potassium, calcium, eGFR, emergency, surgery type, anesthesia method, duration of surgery.

**Figure S6** Sensitivity analysis for RCS curve between TyG, TyG-BMI, and NPAR and the risk of AKI after including patients with complete exposure indices in the exclusion population (n = 8,439). (A) TyG and AKI. (B) TyG-BMI and AKI. (C) NPAR and AKI. AKI: acute kidney injury; NPAR: neutrophil percentage to albumin ratio; RCS: restricted cubic spline; TyG: triglyceride-glucose; TyG-BMI: triglyceride glucose-body mass index. All analyses were adjusted for confounding factors including age, gender, smoking, drinking, diabetes mellitus, hypertension, hyperlipemia, anemia, diuretics, HDL-C, UA, sodium, potassium, calcium, eGFR, emergency, surgery type, anesthesia method, duration of surgery.

**Table S18** Sensitivity analysis for predictive ability of TyG, TyG-BMI, NPAR, TyG/TyG-BMI combined with NPAR for AKI after excluding spinal, osteotomy or deformity correction surgery (remaining patients, n = 3,510)

|  | **AUC (95% CI)** | **Sensitivity** | **Specificity** | ***P*-Value for AUC** | **Cutoff value** |
| --- | --- | --- | --- | --- | --- |
| TyG | 0.592 (0.550 - 0.633)^a^ | 0.552 | 0.606 | <0.001 | 8.678 |
| TyG-BMI | 0.560 (0.516 - 0.603)^a^ | 0.723 | 0.409 | 0.008 | 232.809 |
| NPAR | 0.631 (0.591 - 0.671)^a, b^ | 0.538 | 0.682 | <0.001 | 16.225 |
| TyG combined with NPAR | 0.652 (0.614 - 0.691)^b, c^ | 0.440 | 0.788 | <0.001 | – |
| TyG-BMI combined with NPAR | 0.662 (0.624 - 0.700)^c, d^ | 0.579 | 0.677 | <0.001 | – |

AKI: acute kidney injury; AUC: area under curve; CI: confidence interval; NPAR: neutrophil percentage to albumin ratio; TyG: triglyceride-glucose; TyG-BMI: triglyceride glucose-body mass index. ^a, b, c, d^ The same superscript letters indicate a non-significant difference of AUC.

**Table S19** Sensitivity analysis for predictive ability of TyG, TyG-BMI, NPAR, TyG/TyG-BMI combined with NPAR for AKI after excluding patients with diabetes (remaining patients, n = 4,111)

|  | **AUC (95% CI)** | **Sensitivity** | **Specificity** | ***P*-Value for AUC** | **Cut off value** |
| --- | --- | --- | --- | --- | --- |
| TyG | 0.566 (0.522 - 0.609)^a^ | 0.623 | 0.497 | 0.003 | 8.757 |
| TyG-BMI | 0.558 (0.512 - 0.604)^a^ | 0.740 | 0.382 | 0.014 | 232.777 |
| NPAR | 0.677 (0.634 - 0.719)^b^ | 0.731 | 0.543 | <0.001 | 18.252 |
| TyG combined with NPAR | 0.682 (0.641 - 0.724)^b, c^ | 0.513 | 0.751 | <0.001 | – |
| TyG-BMI combined with NPAR | 0.701 (0.660 - 0.742)^d^ | 0.653 | 0.647 | <0.001 | – |

AKI: acute kidney injury; AUC: area under curve; CI: confidence interval; NPAR: neutrophil percentage to albumin ratio; TyG: triglyceride-glucose; TyG-BMI: triglyceride glucose-body mass index. ^a, b, c, d^ The same superscript letters indicate a non-significant difference of AUC.

**Table S20** Sensitivity analysis for predictive ability of TyG, TyG-BMI, NPAR, TyG/TyG-BMI combined with NPAR for AKI after excluding patients with hyperlipemia (remaining patients, n = 4,616)

|  | **AUC (95% CI)** | **Sensitivity** | **Specificity** | ***P*-Value for AUC** | **Cut off value** |
| --- | --- | --- | --- | --- | --- |
| TyG | 0.587 (0.548 - 0.626)^a^ | 0.594 | 0.555 | <0.001 | 8.757 |
| TyG-BMI | 0.556 (0.516 - 0.597)^a^ | 0.726 | 0.392 | 0.007 | 232.789 |
| NPAR | 0.655 (0.617 - 0.693)^b^ | 0.564 | 0.687 | <0.001 | 16.165 |
| TyG combined with NPAR | 0.667 (0.631 - 0.704)^b, c^ | 0.505 | 0.727 | <0.001 | – |
| TyG-BMI combined with NPAR | 0.682 (0.646 - 0.718)^c, d^ | 0.571 | 0.714 | <0.001 | – |

AKI: acute kidney injury; AUC: area under curve; CI: confidence interval; NPAR: neutrophil percentage to albumin ratio; TyG: triglyceride-glucose; TyG-BMI: triglyceride glucose-body mass index. ^a, b, c, d^ The same superscript letters indicate a non-significant difference of AUC.

**Table S21** Sensitivity analysis for predictive ability of TyG, TyG-BMI, NPAR, TyG/TyG-BMI combined with NPAR for AKI after excluding patients with emergency surgery (remaining patients, n = 4,526)

|  | **AUC (95% CI)** | **Sensitivity** | **Specificity** | ***P*-Value for AUC** | **Cut off value** |
| --- | --- | --- | --- | --- | --- |
| TyG | 0.585 (0.545 - 0.625)^a^ | 0.542 | 0.606 | <0.001 | 8.678 |
| TyG-BMI | 0.561 (0.520 - 0.602)^a^ | 0.723 | 0.404 | 0.004 | 232.809 |
| NPAR | 0.644 (0.606 - 0.683)^b^ | 0.574 | 0.665 | <0.001 | 16.165 |
| TyG combined with NPAR | 0.661 (0.624 - 0.699)^b, c^ | 0.528 | 0.693 | <0.001 | – |
| TyG-BMI combined with NPAR | 0.675 (0.638 - 0.711)^c, d^ | 0.571 | 0.706 | <0.001 | – |

AKI: acute kidney injury; AUC: area under curve; CI: confidence interval; NPAR: neutrophil percentage to albumin ratio; TyG: triglyceride-glucose; TyG-BMI: triglyceride glucose-body mass index. ^a, b, c, d^ The same superscript letters indicate a non-significant difference of AUC.

**Table S22** Sensitivity analysis for predictive ability of TyG, TyG-BMI, NPAR, TyG/TyG-BMI combined with NPAR for AKI after excluding patients with emergency surgery or neutrophil percentage > 75% (remaining patients, n = 3,751)

|  | **AUC (95% CI)** | **Sensitivity** | **Specificity** | ***P*-Value for AUC** | **Cut off value** |
| --- | --- | --- | --- | --- | --- |
| TyG | 0.574 (0.529 - 0.620)^a^ | 0.552 | 0.596 | 0.001 | 8.678 |
| TyG-BMI | 0.585 (0.538 - 0.632)^a^ | 0.708 | 0.447 | <0.001 | 232.809 |
| NPAR | 0.633 (0.587 - 0.679)^b^ | 0.688 | 0.547 | <0.001 | 16.165 |
| TyG combined with NPAR | 0.653 (0.609 - 0.697)^b, c^ | 0.585 | 0.658 | <0.001 | – |
| TyG-BMI combined with NPAR | 0.675 (0.631 - 0.718)^c, d^ | 0.680 | 0.590 | <0.001 | – |

AKI: acute kidney injury; AUC: area under curve; CI: confidence interval; NPAR: neutrophil percentage to albumin ratio; TyG: triglyceride-glucose; TyG-BMI: triglyceride glucose-body mass index. ^a, b, c, d^ The same superscript letters indicate a non-significant difference of AUC.

**Table S23** Sensitivity analysis for predictive ability of TyG, TyG-BMI, NPAR, TyG/TyG-BMI combined with NPAR for AKI after including patients with complete exposure indices in the exclusion population (n = 8,439)

|  | **AUC (95% CI)** | **Sensitivity** | **Specificity** | ***P*-Value for AUC** | **Cut off value** |
| --- | --- | --- | --- | --- | --- |
| TyG | 0.605 (0.566 - 0.643)^a^ | 0.561 | 0.612 | <0.001 | 8.678 |
| TyG-BMI | 0.568 (0.528 - 0.608)^a^ | 0.738 | 0.392 | <0.001 | 232.809 |
| NPAR | 0.715 (0.679 - 0.751)^b^ | 0.667 | 0.685 | <0.001 | 16.166 |
| TyG combined with NPAR | 0.731 (0.696 - 0.765)^c^ | 0.843 | 0.500 | <0.001 | – |
| TyG-BMI combined with NPAR | 0.740 (0.707 - 0.774)^c, d^ | 0.709 | 0.672 | <0.001 | – |

AKI: acute kidney injury; AUC: area under curve; CI: confidence interval; NPAR: neutrophil percentage to albumin ratio; TyG: triglyceride-glucose; TyG-BMI: triglyceride glucose-body mass index. ^a, b, c, d^ The same superscript letters indicate a non-significant difference of AUC.

**Figure S7** Sensitivity analysis for ROC curves between TyG, TyG-BMI, NPAR, TyG/TyG-BMI combined with NPAR and AKI after excluding spinal, osteotomy or deformity correction surgery (remaining patients, n = 3,510). ^*^The AUC was significantly higher than that of TyG alone. ^#^The AUC was significantly higher than that of TyG-BMI and NPAR alone. AKI: acute kidney injury; AUC: area under the curve; NPAR: neutrophil percentage to albumin ratio; ROC: receiver operating characteristic; TyG: triglyceride-glucose; TyG-BMI: triglyceride glucose-body mass index.

**Figure S8** Sensitivity analysis for ROC curves between TyG, TyG-BMI, NPAR, TyG/TyG-BMI combined with NPAR and AKI after excluding patients with diabetes (remaining patients, n = 4,111). ^*^The AUC was significantly higher than that of TyG alone. ^#^The AUC was significantly higher than that of TyG-BMI and NPAR alone. AKI: acute kidney injury; AUC: area under the curve; NPAR: neutrophil percentage to albumin ratio; ROC: receiver operating characteristic; TyG: triglyceride-glucose; TyG-BMI: triglyceride glucose-body mass index.

**Figure S9** Sensitivity analysis for ROC curves between TyG, TyG-BMI, NPAR, TyG/TyG-BMI combined with NPAR and AKI after excluding patients with hyperlipemia (remaining patients, n = 4,616). ^*^The AUC was significantly higher than that of TyG alone. ^#^The AUC was significantly higher than that of TyG-BMI and NPAR alone. AKI: acute kidney injury; AUC: area under the curve; NPAR: neutrophil percentage to albumin ratio; ROC: receiver operating characteristic; TyG: triglyceride-glucose; TyG-BMI: triglyceride glucose-body mass index.

**Figure S10** Sensitivity analysis for ROC curves between TyG, TyG-BMI, NPAR, TyG/TyG-BMI combined with NPAR and AKI after excluding patients with emergency surgery (remaining patients, n = 4,526). ^*^The AUC was significantly higher than that of TyG alone. ^#^The AUC was significantly higher than that of TyG-BMI and NPAR alone. AKI: acute kidney injury; AUC: area under the curve; NPAR: neutrophil percentage to albumin ratio; ROC: receiver operating characteristic; TyG: triglyceride-glucose; TyG-BMI: triglyceride glucose-body mass index.

**Figure S11** Sensitivity analysis for ROC curves between TyG, TyG-BMI, NPAR, TyG/TyG-BMI combined with NPAR and AKI after excluding patients with emergency surgery and neutrophil percentage > 75% (remaining patients, n = 3,751). ^*^The AUC was significantly higher than that of TyG alone. ^#^The AUC was significantly higher than that of TyG-BMI and NPAR alone. AKI: acute kidney injury; AUC: area under the curve; NPAR: neutrophil percentage to albumin ratio; ROC: receiver operating characteristic; TyG: triglyceride-glucose; TyG-BMI: triglyceride glucose-body mass index.

**Figure S12** Sensitivity analysis for ROC curves between TyG, TyG-BMI, NPAR, TyG/TyG-BMI combined with NPAR and AKI after including patients with complete exposure indices in the exclusion population (n = 8,439). ^*^The AUC was significantly higher than that of TyG-BMI and NPAR alone. ^#^The AUC was significantly higher than that of TyG-BMI and NPAR alone. AKI: acute kidney injury; AUC: area under the curve; NPAR: neutrophil percentage to albumin ratio; ROC: receiver operating characteristic; TyG: triglyceride-glucose; TyG-BMI: triglyceride glucose-body mass index.

**Table S24** Accelerated failure time model of TyG, TyG-BMI, NPAR, TyG/TyG-BMI combined with NPAR with the length of postoperative hospital stay

|  | **Model 1** | ***P* value** |  | **Model 2** | ***P* value** |  | **Model 3** | ***P* value** |
| --- | --- | --- | --- | --- | --- | --- | --- | --- |
|  | **TR (95% CI)** |  |  | **TR (95% CI)** |  |  | **TR (95% CI)** |  |
| TyG (Per SD) | 1.061 (1.043, 1.079) | <0.001 |  | 1.062 (1.045, 1.080) | <0.001 |  | 1.038 (1.020, 1.056) | <0.001 |
| TyG-BMI (Per SD) | 0.974 (0.957, 0.990) | 0.002 |  | 0.981 (0.965, 0.997) | 0.022 |  | 0.986 (0.971, 1.003) | 0.098 |
| NPAR (Per SD) | 1.220 (1.200, 1.240) | <0.001 |  | 1.220 (1.200, 1.241) | <0.001 |  | 1.196 (1.172, 1.221) | <0.001 |
| BMI |  |  |  |  |  |  |  |  |
| Underweight | 1.092 (1.016, 1.173) | 0.016 |  | 1.100 (1.025, 1.181) | 0.008 |  | 1.032 (0.965, 1.104) | 0.355 |
| Normal weight | Reference |  |  | Reference |  |  | Reference |  |
| Overweight | 0.921 (0.885, 0.957) | <0.001 |  | 0.922 (0.887, 0.958) | <0.001 |  | 0.951 (0.917, 0.986) | 0.007 |
| Obesity | 0.922 (0.877, 0.968) | 0.001 |  | 0.951 (0.905, 0.999) | 0.004 |  | 0.972 (0.928, 1.018) | 0.235 |
| TyG combined with NPAR |  |  |  |  |  |  |  |  |
| TyG low level & NPAR low level | Reference |  |  | Reference |  |  | Reference |  |
| TyG high level & NPAR low level | 0.987 (0.944, 1.032) | 0.567 |  | 0.990 (0.947, 1.034) | 0.646 |  | 0.988 (0.946, 1.031) | 0.583 |
| TyG low level & NPAR high level | 1.314 (1.259, 1.372) | <0.001 |  | 1.301 (1.246, 1.359) | <0.001 |  | 1.222 (1.169, 1.278) | <0.001 |
| TyG high level & NPAR high level | 1.471 (1.402, 1.544) | <0.001 |  | 1.464 (1.395, 1.537) | <0.001 |  | 1.323 (1.256, 1.394) | <0.001 |
| TyG-BMI combined with NPAR |  |  |  |  |  |  |  |  |
| TyG-BMI low level & NPAR low level | Reference |  |  | Reference |  |  | Reference |  |
| TyG-BMI high level & NPAR low level | 1.029 (0.982, 1.078) | 0.236 |  | 1.043 (0.995, 1.093) | 0.079 |  | 1.039 (0.993, 1.087) | 0.097 |
| TyG-BMI low level & NPAR high level | 1.422 (1.369, 1.479) | <0.001 |  | 1.416 (1.361, 1.472) | <0.001 |  | 1.308 (1.255, 1.364) | <0.001 |
| TyG-BMI high level & NPAR high level | 1.320 (1.245, 1.398) | <0.001 |  | 1.323 (1.249, 1.401) | <0.001 |  | 1.189 (1.121, 1.261) | <0.001 |

CI: confidence interval; NPAR: neutrophil percentage to albumin ratio; SD: standard deviation; TR: time ratio; TyG: triglyceride-glucose; TyG-BMI: triglyceride glucose-body mass index.

Model 1: unadjusted.

Model 2: adjusted for age, gender, smoking, drinking.

Model 3: further adjusted for diabetes mellitus, hypertension, hyperlipemia, anemia, diuretics, HDL-C, UA, sodium, potassium, calcium, eGFR, emergency, surgery type, anesthesia method, duration of surgery based on Model 2.

**Table S25** Logistic regression analysis for the association of TyG, TyG-BMI, and NPAR with ICU admission

|  | **Model 1** | ***P* value** |  | **Model 2** | ***P* value** |  | **Model 3** | ***P* value** |
| --- | --- | --- | --- | --- | --- | --- | --- | --- |
|  | **OR (95% CI)** |  |  | **OR (95% CI)** |  |  | **OR (95% CI)** |  |
| TyG |  |  |  |  |  |  |  |  |
| Q1 | Reference |  |  | Reference |  |  | Reference |  |
| Q2 | 0.940 (0.631, 1.400) | 0.760 |  | 0.885 (0.593, 1.321) | 0.551 |  | 0.917 (0.605, 1.390) | 0.683 |
| Q3 | 1.061 (0.719, 1.563) | 0.767 |  | 0.956 (0.647, 1.414) | 0.823 |  | 1.000 (0.666, 1.503) | 0.999 |
| Q4 | 1.264 (0.869, 1.837) | 0.220 |  | 1.143 (0.784, 1.665) | 0.488 |  | 1.122 (0.747, 1.685) | 0.580 |
| *P* for trend | 1.204 (0.934, 1.552) | 0.153 |  | 1.126 (0.870, 1.457) | 0.367 |  | 1.046 (0.918, 1.193) | 0.500 |
| Per SD | 1.276 (1.046, 1.557) | 0.016 |  | 1.203 (0.982, 1.473) | 0.074 |  | 1.126 (0.978, 1.297) | 0.100 |
| TyG-BMI |  |  |  |  |  |  |  |  |
| Q1 | Reference |  |  | Reference |  |  | Reference |  |
| Q2 | 0.895 (0.614, 1.305) | 0.565 |  | 0.854 (0.585, 1.247) | 0.414 |  | 0.903 (0.607, 1.344) | 0.615 |
| Q3 | 0.860 (0.588, 1.259) | 0.439 |  | 0.800 (0.546, 1.173) | 0.253 |  | 0.845 (0.563, 1.269) | 0.418 |
| Q4 | 0.912 (0.626, 1.327) | 0.629 |  | 0.892 (0.612, 1.302) | 0.555 |  | 0.969 (0.646, 1.452) | 0.878 |
| *P* for trend | 0.999 (0.995, 1.003) | 0.617 |  | 0.999 (0.995, 1.003) | 0.530 |  | 0.983 (0.862, 1.121) | 0.803 |
| Per SD | 1.002 (0.998, 1.005) | 0.311 |  | 1.002 (0.998, 1.005) | 0.346 |  | 1.097 (0.953, 1.262) | 0.197 |
| NPAR |  |  |  |  |  |  |  |  |
| Q1 | Reference |  |  | Reference |  |  | Reference |  |
| Q2 | 1.085 (0.619, 1.901) | 0.775 |  | 1.056 (0.602, 1.851) | 0.850 |  | 1.034 (0.586, 1.825) | 0.908 |
| Q3 | 2.043 (1.243, 3.358) | 0.005 |  | 1.963 (1.190, 3.237) | 0.008 |  | 1.996 (1.197, 3.328) | 0.008 |
| Q4 | 5.606 (3.591, 8.751) | <0.001 |  | 5.115 (3.237, 8.083) | <0.001 |  | 5.209 (3.141, 8.638) | <0.001 |
| *P* for trend | 1.203 (1.161, 1.248) | <0.001 |  | 1.193 (1.149, 1.238) | <0.001 |  | 1.868 (1.578, 2.210) | <0.001 |
| Per SD | 1.120 (1.098, 1.143) | <0.001 |  | 1.115 (1.092, 1.139) | <0.001 |  | 1.672 (1.445, 1.935) | <0.001 |
| BMI |  |  |  |  |  |  |  |  |
| Underweight | 1.528 (0.935, 2.498) | 0.091 |  | 1.539 (0.939, 2.523) | 0.087 |  | 1.443 (0.851, 2.447) | 0.174 |
| Normal weight | Reference |  |  | Reference |  |  | Reference |  |
| Overweight | 0.853 (0.615, 1.184) | 0.343 |  | 0.854 (0.615, 1.186) | 0.347 |  | 0.885 (0.628, 1.246) | 0.483 |
| Obesity | 1.186 (0.814, 1.727) | 0.375 |  | 1.240 (0.848, 1.814) | 0.267 |  | 1.330 (0.891, 1.985) | 0.163 |
| Per SD | 1.010 (0.884, 1.156) | 0.879 |  | 1.021 (0.891, 1.170) | 0.764 |  | 1.062 (0.924, 1.222) | 0.396 |

CI: confidence interval; ICU: intensive care unit; NPAR: neutrophil percentage to albumin ratio; OR: odds ratio; SD: standard deviation; TyG: triglyceride-glucose; TyG-BMI: triglyceride glucose-body mass index.

Model 1: unadjusted.

Model 2: adjusted for age, gender, smoking, drinking.

Model 3: further adjusted for diabetes mellitus, hypertension, hyperlipemia, anemia, diuretics, HDL-C, UA, sodium, potassium, calcium, eGFR, emergency, surgery type, anesthesia method, duration of surgery based on Model 2.

**Table S26** Logistic regression analysis for the association of TyG/TyG-BMI combined with NPAR with ICU admission

|  | **Model 1** | ***P* value** |  | **Model 2** | ***P* value** |  | **Model 3** | ***P* value** |
| --- | --- | --- | --- | --- | --- | --- | --- | --- |
|  | **OR (95% CI)** |  |  | **OR (95% CI)** |  |  | **OR (95% CI)** |  |
| TyG combined with NPAR |  |  |  |  |  |  |  |  |
| TyG low level & NPAR low level | Reference |  |  | Reference |  |  | Reference |  |
| TyG high level & NPAR low level | 0.816 (0.478, 1.396) | 0.458 |  | 0.768 (0.449, 1.316) | 0.337 |  | 0.729 (0.422, 1.262) | 0.259 |
| TyG low level & NPAR high level | 2.740 (1.835, 4.091) | <0.001 |  | 2.484 (1.649, 3.741) | <0.001 |  | 2.312 (1.500, 3.564) | <0.001 |
| TyG high level & NPAR high level | 4.380 (2.933, 6.540) | <0.001 |  | 3.868 (2.565, 5.832) | <0.001 |  | 3.493 (2.234, 5.460) | <0.001 |
| TyG-BMI combined with NPAR |  |  |  |  |  |  |  |  |
| TyG-BMI low level & NPAR low level | Reference |  |  | Reference |  |  | Reference |  |
| TyG-BMI high level & NPAR low level | 0.787 (0.440, 1.409) | 0.421 |  | 0.767 (0.428, 1.374) | 0.372 |  | 0.671 (0.366, 1.229) | 0.196 |
| TyG-BMI low level & NPAR high level | 3.187 (2.226, 4.563) | <0.001 |  | 2.887 (1.999, 4.170) | <0.001 |  | 2.583 (1.741, 3.833) | <0.001 |
| TyG-BMI high level & NPAR high level | 4.228 (2.741, 6.524) | <0.001 |  | 3.917 (2.526, 6.075) | <0.001 |  | 3.432 (2.155, 5.465) | <0.001 |

CI: confidence interval; ICU: intensive care unit; NPAR: neutrophil percentage to albumin ratio; OR: odds ratio; TyG: triglyceride-glucose; TyG-BMI: triglyceride glucose-body mass index.

Model 1: unadjusted.

Model 2: adjusted for age, gender, smoking, drinking.

Model 3: further adjusted for diabetes mellitus, hypertension, hyperlipemia, anemia, diuretics, HDL-C, UA, sodium, potassium, calcium, eGFR, emergency, surgery type, anesthesia method, duration of surgery based on Model 2.

**Table S27** Association of BMI with postoperative AKI

|  | **Model 1** | ***P* value** |  | **Model 2** | ***P* value** |  | **Model 3** | ***P* value** |
| --- | --- | --- | --- | --- | --- | --- | --- | --- |
|  | **OR (95% CI)** |  |  | **OR (95% CI)** |  |  | **OR (95% CI)** |  |
| BMI |  |  |  |  |  |  |  |  |
| Underweight | 1.619 (0.977, 2.684) | 0.061 |  | 1.545 (0.921, 2.593) | 0.100 |  | 1.410 (0.819, 2.430) | 0.215 |
| Normal weight | Reference |  |  | Reference |  |  | Reference |  |
| Overweight | 1.188 (0.865, 1.631) | 0.288 |  | 1.208 (0.876, 1.665) | 0.250 |  | 1.348 (0.961, 1.892) | 0.084 |
| Obesity | 1.593 (1.109, 2.290) | 0.012 |  | 1.725 (1.190, 2.503) | 0.004 |  | 1.736 (1.167, 2.582) | 0.007 |
| Per SD | 1.115 (0.981, 1.268) | 0.096 |  | 1.141 (1.001, 1.300) | 0.049 |  | 1.153 (1.008, 1.320) | 0.039 |

AKI: acute kidney injury; BMI: body mass index; CI: confidence interval; OR: odds ratio; SD: standard deviation.

Model 1: unadjusted.

Model 2: adjusted for age, gender, smoking, drinking.

Model 3: further adjusted for diabetes mellitus, hypertension, hyperlipemia, anemia, diuretics, HDL-C, UA, sodium, potassium, calcium, eGFR, emergency, surgery type, anesthesia method, duration of surgery based on Model 2.
